# Tutorial on compressed ultrafast photography

**DOI:** 10.1117/1.JBO.29.S1.S11524

**Published:** 2024-01-30

**Authors:** Yingming Lai, Miguel Marquez, Jinyang Liang

**Affiliations:** Université du Québec, Institut National de la Recherche Scientifique, Centre Énergie Matériaux Télécommunications, Laboratory of Applied Computational Imaging, Varennes, Québec, Canada

**Keywords:** single-shot computational imaging, coded optical imaging, compressed sensing, streak imaging, image reconstruction techniques, transient biomedical phenomena

## Abstract

**Significance:**

Compressed ultrafast photography (CUP) is currently the world’s fastest single-shot imaging technique. Through the integration of compressed sensing and streak imaging, CUP can capture a transient event in a single camera exposure with imaging speeds from thousands to trillions of frames per second, at micrometer-level spatial resolutions, and in broad sensing spectral ranges.

**Aim:**

This tutorial aims to provide a comprehensive review of CUP in its fundamental methods, system implementations, biomedical applications, and prospect.

**Approach:**

A step-by-step guideline to CUP’s forward model and representative image reconstruction algorithms is presented with sample codes and illustrations in Matlab and Python. Then, CUP’s hardware implementation is described with a focus on the representative techniques, advantages, and limitations of the three key components—the spatial encoder, the temporal shearing unit, and the two-dimensional sensor. Furthermore, four representative biomedical applications enabled by CUP are discussed, followed by the prospect of CUP’s technical advancement.

**Conclusions:**

CUP has emerged as a state-of-the-art ultrafast imaging technology. Its advanced imaging ability and versatility contribute to unprecedented observations and new applications in biomedicine. CUP holds great promise in improving technical specifications and facilitating the investigation of biomedical processes.

## 1 Introduction

Optical imaging of transient events in their actual time of occurrence exerts compelling scientific significance and practical merits.^1^ Occurring in two-dimensional (2D) space and at femtosecond ($1\;\mathrm{fs}=10^{-15}\;s$) to microsecond ($1\;\mu s=10^{-6}\;s$) time scales, these transient events reflect many important fundamental mechanisms in biology.^2^^–^^4^ However, many transient phenomena are either nonrepeatable or difficult to reproduce. Examples include the spontaneous synaptic activities,^5^ nanoparticles’ luminescence lifetime at different temperatures,^6^ and light scattering in living tissue.^7^ Under these circumstances, the conventional pump–probe methods, requiring numerous repeatable experiments, are inapplicable. Meanwhile, the pump–probe approaches sense photons’ time-of-arrival using complex apparatus to perform time-consuming scanning in either space or time. In these cases, even if the transient phenomena are reproducible, these methods would suffer from substantial inaccuracy due to experimental perturbation and low productivity due to the events’ low occurrence rates.

Single-shot ultrafast optical imaging techniques^8^^,^^9^ can overcome these limitations by capturing the entire dynamic process in real time (i.e., in the actual duration of the event’s occurrence) without repeating measurements. Benefiting from advancements in optoelectronics, laser science, information theory, and computational techniques, single-shot ultrafast optical imaging has become a burgeoning research field in the past decade. Thus far, the mainstream techniques can be generally categorized into the domains of active illumination and passive detection. For the former, temporal information of a dynamic scene is mapped into an optical marker (e.g., spectrum and spatial frequency) of one or multiple ultrashort probe pulses. On the detection side, appropriate devices and methods (e.g., color filter and spatial Fourier transformation) are used to extract the corresponding optical marker, which deduces the scene’s evolution. These active-illumination-based approaches feature femtosecond temporal resolution by leveraging ultrashort durations of ultrafast probe pulses and provide high sensitivity by being compatible with advanced cameras based on the charge-coupled device (CCD) or complementary metal–oxide semiconductor (CMOS) technology. Nonetheless, they cannot capture the self-luminescence scenes, including dynamic scattering,^10^ photoluminescence intensity decay,^11^ and plasma emission.^12^ Passive detection can overcome this limitation. In this category, receive-only ultrafast detectors are used to record the emitted and/or scattered photons from the dynamic scene. Various mechanisms, including Kerr-effect-based time gating,^13^ deflection of moving photoelectrons by a varying electrical field,^14^ and charge transfer in a series of registers,^15^ have been used to provide ultrahigh temporal resolution. Meanwhile, the inferior bandwidth of electronics to its optical counterpart caps the ultimate imaging speed of these passive-detection approaches lower to the active-illumination modalities. Overall, the active illumination and passive detection approaches often carry highly complementary technical specifications. Altogether, they incessantly expand the human vision to see previous inaccessible events.

Among existing techniques, compressed ultrafast photography (CUP) has emerged as a potent single-shot ultrafast optical imaging modality.^16^ Invented in 2014 in Dr. Lihong V. Wang’s laboratory,^17^ CUP innovatively synergizes compressed sensing (CS) with streak imaging. Leveraging the sparsity existing in the targeted scenes, the operation of this hybrid approach includes physical data acquisition followed by computational image reconstruction.^18^^,^^19^ In data acquisition, the light from a 2D dynamic scene is recorded in one or more snapshots in a single shot via a CS paradigm containing spatial encoding, temporal shearing, and spatiotemporal integration. Different from conventional ultrafast imaging, the acquired snapshot often bears no resemblance to the scene. Then, the snapshot is input into an algorithm to retrieve the movie of the target dynamic scene by solving a minimization problem.^20^

CUP provides many attractive conceptual novelties and practical advantages. First, the spatial encoding and temporal shearing operations allow a mixture of information between time and space, which enables CUP to have a large sequence depth (i.e., the number of frames in each recorded movie) compared with other single-shot ultrafast imaging systems based on spatial frequency multiplexing,^21^^–^^24^ spectral filtering,^25^^–^^30^ and beam splitting.^31^^–^^34^ Meanwhile, it overcomes the limitations in sensing dimension in conventionally regarded one-dimensional (1D) high-speed sensors.^14^^,^^35^ Compared with ultrafast CCD sensors that have a low fill factor, CUP uses spatiotemporal multiplexing to effectively enhance the light throughput in data acquisition, which improves the feasibility of image reconstruction.^36^ It is compatible with many scientific-grade CCD/CMOS sensors without interrupting their normal operations, which retains their responsive spectrum and sensitivity while endowing them with ultrahigh speeds.^37^ Second, its generic sensing paradigm can be embodied in both active-illumination and passive-detection schemes. Each major operation (i.e., spatial encoding, temporal shearing, and spatiotemporal integration) can be optically realized by various devices, indicating high design flexibility, multi-spatiotemporal-scale imaging ability, and broad spectral coverage. Third, computational image reconstruction, as an indispensable step in CUP’s operation, lifts certain burdens in system design from hardware. Advances in CS,^38^ machine learning,^39^ and information theory^40^ can be directly implemented in CUP’s image reconstruction. Finally, CUP exhibits light throughput advantages by capturing information in two spatial dimensions and time simultaneously in a single exposure. In contrast, the multiple-shot methods can only collect information from a column (from point scanning) or a slice (from line scanning) of the datacube.^41^ Meanwhile, distinguished from single-shot framing (or mapping) photography,^30^^,^^34^^,^^42^ CUP maintains time continuity in data acquisition, which further enhances the amount of acquirable information.^18^

Because of its unprecedented imaging ability, CUP immediately became a research focus since its invention. New designs in hardware and innovative development of image reconstruction are being reported frequently. New applications in biomedicine, physics, and engineering are highlighted. Comprehensive reviews of CUP can be found in the literature.^16^^,^^43^ Other reviews of CUP are included in the surveys of ultrafast imaging technologies.^1^^,^^8^^,^^9^^,^^18^^,^^44^^–^^48^ However, thus far, there has not been a practical guide for developing CUP systems using an anatomy fashion. Thus, in this tutorial, we first review the operating principle of CUP with simulation examples (in Matlab and Python) to guide readers on how to generate compressively recorded snapshots from a spatiotemporal datacube using the forward model as well as how to reconstruct the spatiotemporal datacube from the snapshots using representative methods in analytical-modeling-based approach and machine learning. Then, we will provide an extensive survey of existing methods for each of the major operations in CUP’s sensing paradigm—spatial encoding, temporal shearing, and spatiotemporal integration. Afterward, we will discuss the representative applications of CUP in biomedicine. Finally, we summarize CUP’s accomplishments and provide the prospect of its future development.

## 2 Method

**Fig. 1:**
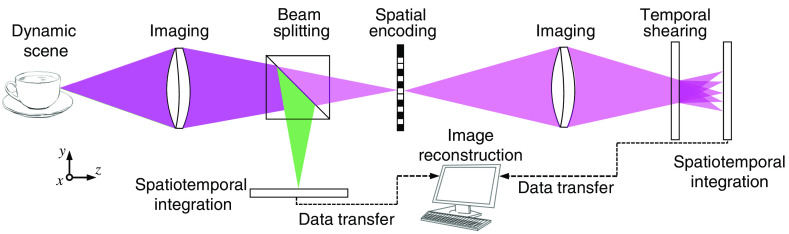
Operating principle of dual-view CUP. The illustration depicts the beam paths for time-sheared and time-unsheared views, represented by magenta and green colors, respectively.

A schematic of dual-view CUP is shown in Fig. 1. In data acquisition, a dynamic scene is imaged by front optics and split into two arms. The transmitted component forms the image of the dynamic scene on a spatial encoder. Unlike many other compressive temporal imaging modalities that use multiple fast-changing patterns during image acquisition,^49^^–^^54^ a single static pattern is used for CUP’s spatial encoder. Then, the frames in the spatially encoded scene are deflected by a temporal shearing unit to different spatial positions along the sweeping direction. Finally, the encoded and sheared scene is spatiotemporally integrated by a sensor, producing a compressive 2D snapshot, which is defined as the time-sheared view and used hereafter in this tutorial. This paradigm to capture the time-sheared view was implemented in the original CUP configuration.^17^ In the ensuing implementations, it was found that a direct capture of a time-integrated snapshot of the dynamic scene could enhance the reconstructed imaging quality.^55^ Defined as the time-unsheared view and used hereafter in this tutorial, this snapshot outlines the region of occurrence of the dynamic scene, which reduces the number of unknowns for image reconstruction and facilitates its convergence to the optimal result. It is particularly useful when the dynamic scene occurs on a static or slowly moving object (e.g., intensity decay of photoluminescence emitted from nanoparticle-labeled cells^56^). It is noted that CUP systems of more than two views have been featured in recent progress to further boost image quality.^57^^–^^66^ For example, lossless-encoding CUP contains the time-unsheared view and two complementary time-sheared views.^58^ Nonetheless, the formation of these views shares similar data acquisition paradigms as the ones described above and thus is not discussed here.

To assist readers in comprehending CUP’s paradigm, in Secs. 2.1 and 2.2, we provide in-depth theoretical derivation and simulation. The presented examples are meticulously designed to use basic features and functions in Matlab (R2020b) and Python (version 3.9). Two versions of Python codes are prepared for readers with different levels of programming experience.

### 2.1 Forward Model

The forward model of CUP formulates the process of recording a three-dimensional [3D; i.e., (x,y,t)] scene to one or a few 2D snapshots. In general, this forward model can be expressed mathematically using either element-wise or matrix-vector notations.

#### 2.1.1 Element-wise notation

Many mathematical and scientific libraries are designed to efficiently handle element-wise operations. The dynamic scene and the binary-valued encoding mask are denoted by $F\in R^{M\times N\times L}$ and $R\in R^{M\times N}$, respectively. M and N represent the data lengths in the two spatial dimensions, and L signifies the data length in time. The discrete output from the sensor for the time-sheared view (hence the subscript “ts”) can be modeled as

$${\left(G_{\mathrm{ts}}\right)}_{i,j}=\overset{L-1}{\underset{l=0}{\sum }}{\overset{¯}{F}}_{i,j,l}{\overset{¯}{R}}_{i,j}+(E_{\mathrm{ts}}{)}_{i,j},$$ (1)

where

$${\overset{¯}{F}}_{i,j,l}=\{\begin{array}{ll} F_{i,j-l,l} & \text{if }l\leq j\leq [N+(l-1)]\\ 0 & \text{otherwise}, \end{array}$$

and

$${\overset{¯}{R}}_{i,j}=\{\begin{array}{ll} R_{i,j-l} & \text{if }l\leq j\leq [N+(l-1)]\\ 0 & \text{otherwise}, \end{array}$$

where ${\overset{¯}{F}}_{i,j,l}$ is the intensity of the (i,j,l)’th element of a right-zero-padded version with a frame-dependent right circular shifting of the dynamic scene’s datacube with $\overset{¯}{F}\in R^{M\times [N+(L-1)]\times L}$. ${\overset{¯}{R}}_{i,j}$ stands for the intensity of the (i,j)’th element a right-zero-padded version with a frame-dependent right circular shifting of the spatial encoder with $\overset{¯}{R}\in R^{M\times [N+(L-1)]}$. $(G_{\mathrm{ts}}{)}_{i,j}$ is the intensity measured on the (i,j)’th element of the sensor with $G_{\mathrm{ts}}\in R^{M\times [N+(L-1)]}$. ${\left(E_{\mathrm{ts}}\right)}_{i,j}$ stands for the noise of the (i,j)’th element in $G_{\mathrm{ts}}$ with $E_{\mathrm{ts}}\in R^{M\times [N+(L-1)]}$.

The discrete output for the time-unsheared view (hence “tu” as the subscript) can be modeled as

$${\left(G_{\mathrm{tu}}\right)}_{i,j}=\overset{L-1}{\underset{l=0}{\sum }}F_{i,j,l}+{\left(E_{\mathrm{tu}}\right)}_{i,j},$$ (2)

where ${\left(G_{\mathrm{tu}}\right)}_{i,j}$ is the intensity measured at the (i,j)’th element of the time-unsheared view with $G_{\mathrm{tu}}\in R^{M\times N}$, and ${\left(E_{\mathrm{tu}}\right)}_{i,j}$ represents the noise in $G_{\mathrm{tu}}$ with $E_{\mathrm{tu}}\in R^{M\times N}$.

**Fig. 2:**
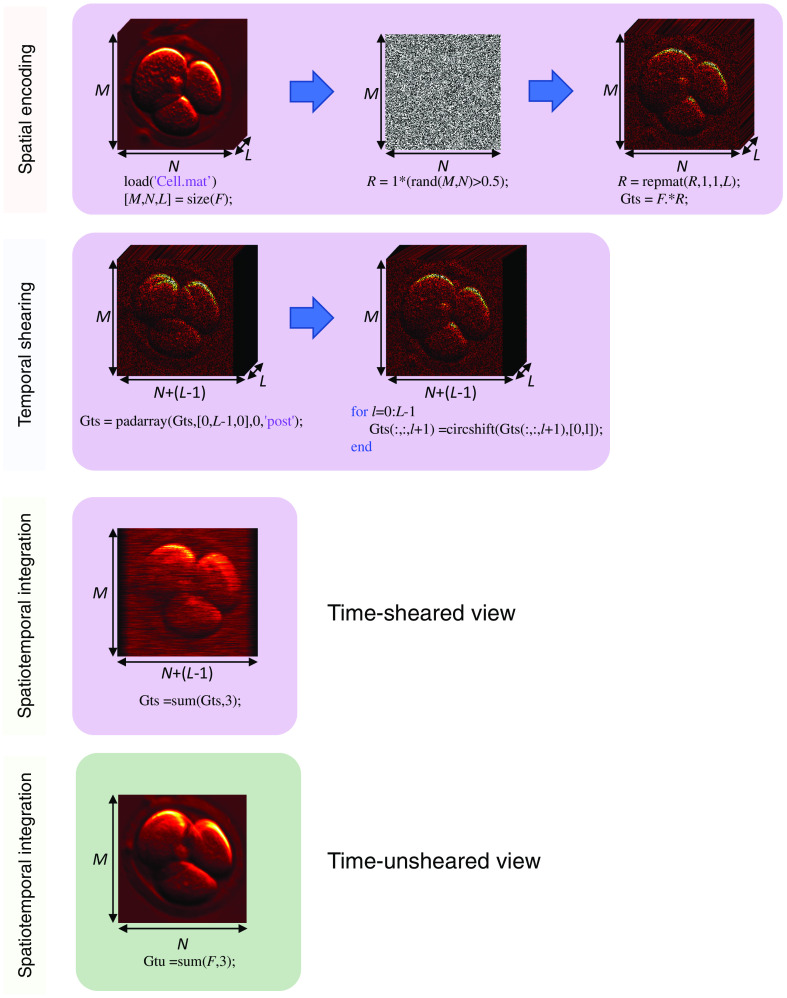
Illustrations of simulating dual-view CUP’s forward model with the element-wise notation using Matlab.

As an example, a Matlab script that simulates dual-view CUP’s forward model, with a linear shearing operator and a pseudorandom binary mask, is shown in Algorithm 1. Moreover, a step-by-step guide with illustrations of the “cell-division” dynamic scene is shown in Fig. 2. The ground truth video was taken from the public “Mouse Embryo Tracking” database^67^ and can be downloaded using the link in Ref. 68.

**Algorithm 1:** Simulating dual-view CUP’s forward model with the element-wise notation using Matlab.

%% Example of dual-view CUP’s sensing process	
% Encoding step (generating R)	
load(‘Cell.mat’)	% Loading the example video – F
[M,N,L] = size(F);	% Calculating the video dimensions % (y,x,t)->(M,N,L)
R = 1*(rand(M,N)>0.5);	% Mask initialization with a % transmittance of ∼50%
R = repmat(R,1,1,L);	
Gts = F.* R;	% Spatial encoding – Hadamard product
Gts = padarray(Gts,[0,L-1,0],0,‘post’);	% Right column zero padding
for l=0:L-1	% Shearing operation
Gts(:,:,l+1) =circshift(Gts(:,:,l+1),[0,l]);	
end	
Gts =sum(Gts,3);	% Integration of time-sheared view
Gtu =sum(F,3);	% Integration of time-unsheared view

#### 2.1.2 Matrix-vector notation

The element-wise notation of CUP’s forward model, despite owning simple expression and easy comprehension, is inherently limited by its sequential execution. This characteristic engenders surplus calculations within specific functions when the modeling is subjected to extensive datasets or algorithms mandating intricate computations—such as matrix inversion, matrix factorization, eigenvalue decomposition, and low-rank approximation. Thus, most practices use matrix-vector operations by converting F into vector $f\in R^{n\times 1}$, where n=M·N·L. In this way, CUP’s forward model can be expressed by matrix multiplication, which can be computed by powerful linear algebra methods to concisely formulate solutions.

In particular, dual-view CUP’s forward model, by following a matrix-vector representation, can be expressed as

$$\begin{array}{c} g=[\begin{array}{c} g_{\mathrm{ts}}\\ g_{\mathrm{tu}} \end{array}]=\Phi f=[\begin{array}{c} T_{\mathrm{ts}}\mathrm{SC}\\ T_{\mathrm{tu}} \end{array}]f, \end{array}$$ (3)

where, $g_{\mathrm{ts}}\in R^{m_{\mathrm{ts}}\times 1}$ and $g_{\mathrm{tu}}\in R^{m_{\mathrm{tu}}\times 1}$ are the vectorial version of the time-sheared view and the time-unsheared view with sizes $m_{\mathrm{ts}}=M\cdot [N+(L-1)]$ and $m_{\mathrm{tu}}=M\cdot N$, respectively. $g\in R^{m\times 1}$ is the vectorial version of the concatenated two views with a size $m=m_{\mathrm{ts}}+m_{\mathrm{tu}}$. $\Phi \in R^{m\times n}$ is the dual-view CUP’s sensing matrix. $C\in R^{n\times n}$ is the spatial encoding matrix. $S\in R^{(m_{\mathrm{ts}}\cdot L)\times n}$ is the temporal shearing matrix. $T_{\mathrm{ts}}\in R^{m_{\mathrm{ts}}\times (m_{\mathrm{ts}}\cdot L)}$ and $T_{\mathrm{tu}}\in R^{m_{\mathrm{tu}}\times n}$ are the spatiotemporal integration matrices of the time-sheared view and the time-unsheared view, respectively. $\Phi_{\mathrm{ts}}=T_{\mathrm{ts}}\mathrm{SC}\in R^{m_{\mathrm{ts}}\times n}$ is also defined as the time-sheared sensing matrix.

The entries of C, S, $T_{\mathrm{ts}}$, and $T_{\mathrm{tu}}$ are given as

$$C_{i,j}=\{\begin{array}{ll} r_{v} & \text{if }i=j\\ 0 & \text{otherwise} \end{array},$$ (4)

$$S_{i,j}=\{\begin{array}{ll} 1 & \text{if }i=j+M\cdot L\cdot \lfloor \frac{j}{M\cdot N}\rfloor \\ 0 & \text{otherwise}, \end{array}$$ (5)

$$T_{\mathrm{ts}}=1_{L}^{T}⊗I_{m_{\mathrm{ts}}\times m_{\mathrm{ts}},}$$ (6)

$$T_{\mathrm{tu}}=1_{L}^{T}⊗I_{m_{\mathrm{tu}}\times m_{\mathrm{tu}}}.$$ (7)

In Eq. (4), v=mod(j,M·N) with v∈W, and $r_{v}\in \{0,1\}$ is the value at the v’th position of $r\in R^{M\cdot N\times 1}$, which is the vectorial version of the encoding mask R. In Eqs. (6) and (7), $1_{L}\in R^{L\times 1}$ is an all-one vector. I is the identity matrix. The matrix $T_{\mathrm{tu}}$ has a structure similar to $T_{\mathrm{ts}}$ (i.e., a horizontal concatenation of identity matrices) but with a shorter diagonal dimension. Thus, the sensing matrix $\Phi =[\begin{array}{c} \Phi_{\mathrm{ts}}\\ T_{\mathrm{tu}} \end{array}]$ can be directly defined as

$$\Phi_{i,j}=\{\begin{array}{ll} r_{v} & \mathrm{if}\;i=v+M\cdot \left\lfloor \frac{j}{M\cdot N}\right\rfloor \\ 1 & \text{if }i=v+m_{\mathrm{ts}}\\ 0 & \text{otherwise} \end{array}.$$ (8)

**Fig. 3:**
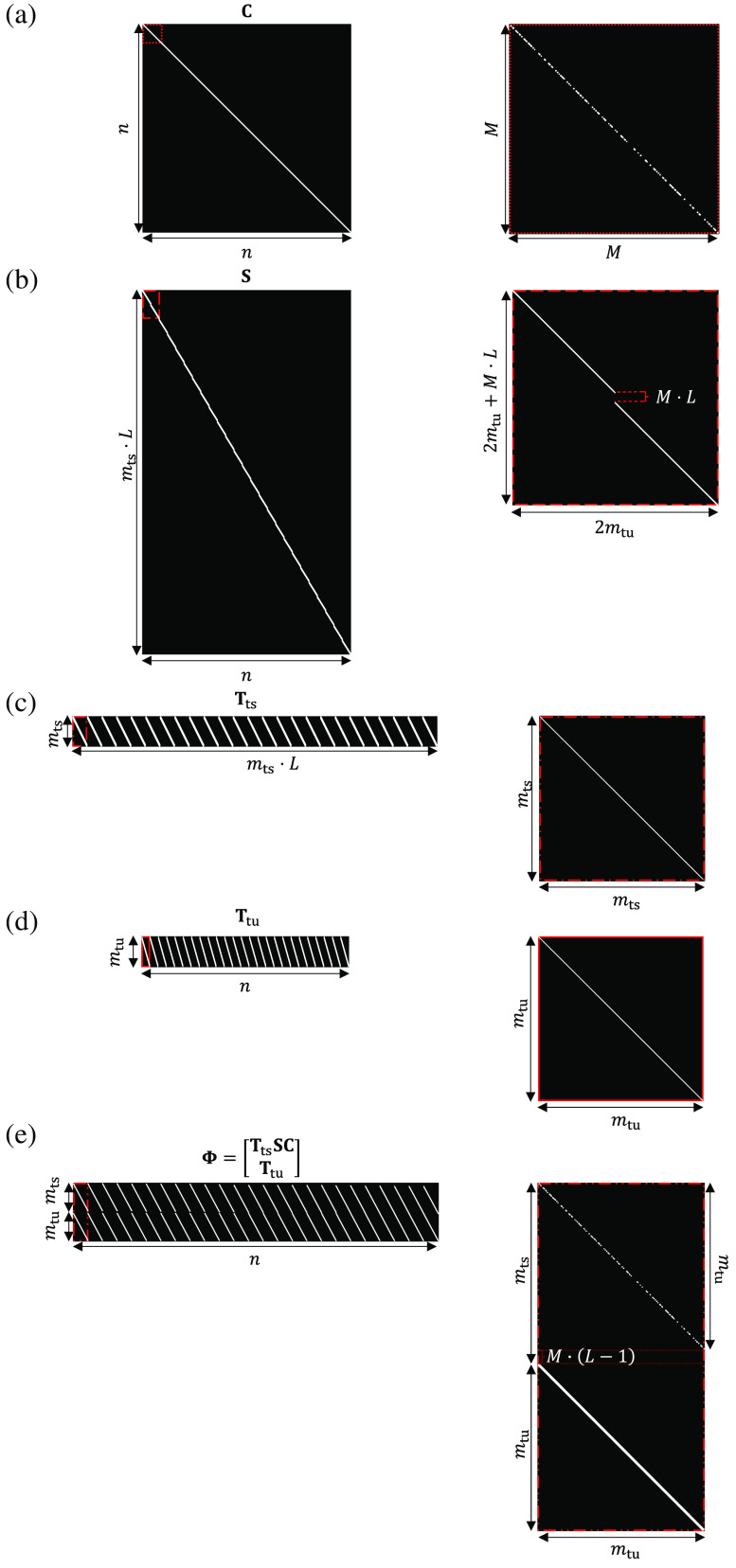
Construction of the matrices of dual-view CUP’s operation using Matlab. (a) Spatial encoding matrix. (b) Temporal shearing matrix. (c) Spatiotemporal integration matrix for the time-sheared view. (d) Spatiotemporal integration matrix for the time-unsheared view. (e) Sensing matrix of dual-view CUP. Insets: Zoomed-in views of local regions (indicated by the red boxes with different line types).

A Matlab script for constructing the matrices C, S, $T_{\mathrm{ts}}$, $T_{\mathrm{tu}}$, and Φ is presented in Algorithm 2. In this example, M×N×L=256×256×25  pixels. The sensing matrix of the time-sheared view $\Phi_{\mathrm{ts}}$ is created by assembling a series of diagonal patterns that cyclically repeat along the horizontal direction, shifting downward by M rows following each iteration. The sensing matrix Φ, illustrated schematically in Fig. 3, is created by vertically concatenating $\Phi_{\mathrm{ts}}$ with $T_{\mathrm{tu}}$.

**Algorithm 2:** Programming matrices of dual-view CUP’s operation using Matlab.

clear all; close all; clc
M = 256; N = 256; L = 25;
R = 1*(rand(M,N)>0.5);
R = repmat(R,1,1,L);
%%
n = M*N*L;
mts = M*(N+L-1);
mtu = M*N;
%% Encoding matrix (C)
i = 0:n-1;
j = 0:n-1;
C = sparse(i+1,j+1,R(:),n,n);
%% Shearing matrix (S)
j = 0:n-1;
i = j+(M*L)*floor(j/(M*N));
S = sparse(i+1,j+1,1,mts*L,n);
%% Integration matrices (Ts and Tu)
Tts = kron(ones(1,L),speye(mts,mts));
Ttu = kron(ones(1,L),speye(mtu,mtu));
%% CUP sensing matrix (\Phi)
Phi = [((Tts*S)*C)’,Ttu’]’;

### 2.2 Image Reconstruction

After data acquisition, the captured 2D snapshots (i.e., $G_{\mathrm{ts}}$ and $G_{\mathrm{tu}}$) are input to an algorithm to reconstruct the dynamic scene. To date, analytical-modeling-based algorithms are dominantly used in CUP’s reconstruction because they can incorporate prior knowledge about the imaging system and the underlying physics of light propagation,^69^^–^^74^ leading to accurate reconstructions. Before getting into sophisticated analytical-modeling-based reconstruction algorithms for CUP, let us analyze the structure of a basic optimization problem:

$$\overset{˜}{f}=\arg\;\underset{f}{\min}\;\frac{1}{2}{\left\|g-\Phi f\right\|}_{2}^{2}+\lambda \varphi (f),$$ (9)

where ${\left\|\cdot \right\|}_{2}^{2}$ is the $\ell_{2}$-norm, λ>0 is a regularization parameter, $\varphi (\cdot ):R^{n}\to R$ is a convex and smooth function, and $\overset{˜}{f}\in R^{n\times 1}$ represents the reconstruction.

Various reconstruction algorithms^75^^–^^78^ are developed based on Eq. (9). A popular choice, especially in the early stage of CUP’s development, is the two-step iterative shrinkage/thresholding (TwIST) algorithm.^78^ The regularizer, φ(f), can be set to various forms, including $\|{\Psi f\|}_{1}$ and ${\left\|f\right\|}_{\mathrm{TV}}$, where ${\left\|\cdot \right\|}_{1}$ represents the $\ell_{1}$-norm, $\Psi \in R^{n\times n}$ is an arbitrary representation basis matrix, and ${\left\|\cdot \right\|}_{\mathrm{TV}}$ represents the total-variation (TV) regularization.^79^ The TwIST algorithm combines the shrinkage operation used in iterative soft-thresholding algorithms with a correction step that enforces fidelity to the measurements. It exploits the sparsity naturally embedded in the transient scene via the regularizer. In particular, the $\ell_{1}$-norm requires prior knowledge about the scene to select an adequate representation basis. The TV-norm uses spatiotemporal correlation by removing low variations between neighboring pixels. These characteristics enable the TwIST algorithm to efficiently recover a transient scene from an underdetermined measurement.

Later, more advanced image reconstruction algorithms are developed based on the paradigm of alternating direction method of multipliers (ADMM),^69^^,^^80^^–^^83^ which sets $\varphi (f)={\left\|f-f^{\left(k\right)}\right\|}_{2}^{2}$, where $f^{\left(k\right)}$ stands for the k’th reconstruction with k={0,…,K−1} and K∈N as the total number of the algorithm’s iterations. The ADMM has gained increasing popularity due to its flexibility to customize the optimization steps by incorporating additional constraints (e.g., noise reduction algorithms or neural-network approaches), which is hence selected for this tutorial.

#### 2.2.1 Analytical-modeling-based approaches

The ADMM accomplishes distributed convex optimization using a divide-and-conquer approach, where a global problem is split into a few subproblems.^84^ Leveraging dual decomposition and the augmented Lagrangian (AL) methods for constrained optimization,^77^ the ADMM solves problems expressed by the following form:

minimize  γ(f)+ψ(z)subject to  Df+Bz=b, (10)

where {γ,ψ} are convex functions. In the equality constraint, D and B are arbitrary matrices that establish a linear relationship between the objective variable (i.e., f) and an auxiliary variable (i.e., z) that works as prior information. The variable b represents the limits (or bounds) for the equality constraint. For instance, in dual-view CUP, an equality constraint can be imposed by setting $D=T_{\mathrm{tu}}$, B=−I, $z=g_{\mathrm{tu}}$, and b=0.

Equation (10) can be solved using the method of Lagrange multipliers, which is a mathematical technique used to optimize a function subject to equality constraints. For Eq. (10), its Lagrangian function is defined as

$$L(f,z,\nu )=\gamma (f)+\psi (z)+\nu^{T}(\mathrm{Df}+\mathrm{Bz}-b),$$ (11)

where ν is the Lagrange multiplier. As a scaling factor, ν enables constructing, from Eq. (10), an unconstrained optimization function, in which the gradients of both the objective function and the constraint function are proportional to each other at the optimal solution.^84^ Then, Eq. (11) is rewritten as^85^

$$\underset{f,z}{\min}\;\underset{\nu }{\max}\;L(f,z,\nu ).$$ (12)

Equation (12) is maximized when ν→+∞ unless Df+Bz−b=0. By converting the maximization problem into a minimization problem [i.e., $\underset{\nu }{\max}\;L(f,z,\nu )=\underset{\nu }{\min}-L(f,z,\nu )$] (Ref. 86) and using a proximal term^87^ to solve the new minimization problem, Eq. (12) results in

$$\underset{f,z}{\min}(\underset{\nu }{\arg\;\min}-L(f,z,\nu )+\frac{1}{2\rho }{\left\|\nu -\overset{¯}{\nu }\right\|}_{2}^{2}),$$ (13)

where ρ>0 is the penalty parameter, and $\overset{¯}{\nu }$ is a previous estimate of ν. Note that the “argmin” in Eq. (13) is now a convex quadratic function with the trivial solution $\nu =\overset{¯}{\nu }+\rho (\mathrm{Df}+\mathrm{Bz}-b)$. By inserting this trivial solution into Eq. (12), the AL-based dual-problem can be obtained as

$$\underset{f,z}{\mathrm{argmin}}\;\gamma (f)+\psi (z)+{\overset{¯}{\nu }}^{T}(\mathrm{Df}+\mathrm{Bz}-b)+\frac{\rho }{2}{\left\|\mathrm{Df}+\mathrm{Bz}-b\right\|}_{2}^{2}.$$ (14)

Finally, Eq. (14) can be split into three optimization problems:

$$f^{\left(k+1\right)}≜\arg\;\underset{f}{\min}\;\gamma (f)+(\nu^{\left(k\right)}{)}^{T}(\mathrm{Df}+{\mathrm{Bz}}^{\left(k\right)}-b)+\frac{\rho }{2}{\left\|\mathrm{Df}+{\mathrm{Bz}}^{\left(k\right)}-b\right\|}_{2}^{2},$$ (15)

$$z^{\left(k+1\right)}≜\arg\;\underset{z}{\min}\;\psi (z)+(\nu^{\left(k\right)}{)}^{T}({\mathrm{Df}}^{\left(k+1\right)}+\mathrm{Bz}-b)+\frac{\rho }{2}{\left\|{\mathrm{Df}}^{\left(k+1\right)}+\mathrm{Bz}-b\right\|}_{2}^{2},$$ (16)

$$\nu^{\left(k+1\right)}≜\nu^{\left(k\right)}+\rho ({\mathrm{Df}}^{\left(k+1\right)}+{\mathrm{Bz}}^{\left(k+1\right)}-b),$$ (17)

where $\left\{\nu^{\left(k+1\right)},\nu^{\left(k\right)}\right\}$ are the equal expressions of $\left\{\nu ,\overset{¯}{\nu }\right\}$, respectively. In this strategy, Eqs. (15)–(17) are solved in an alternating and iterative form to find a point that belongs to the intersection of the two closed convex solution sets. Here, for each step, all the parameters are fixed except the optimization variables [e.g., f in Eq. (15) and z in Eq. (16)]. Then, by repeatedly projecting the updated variables onto each set, the algorithm converges toward a point that satisfies the constraints of all the sets simultaneously.

After defining the core structure of the ADMM algorithm, the following sections discuss two popular variants of the ADMM in image processing.^84^

##### Scaled form ADMM

The scaled form^84^ can be obtained using the equality $\nu^{T}r+(\rho /2){\left\|r\right\|}_{2}^{2}=\frac{\rho }{2}{\left\|r+w\right\|}_{2}^{2}-\frac{\rho }{2}{\left\|w\right\|}_{2}^{2}$ (Ref. 86) with r=Df+Bz−b and the scaled Lagrange multiplier $w=\frac{1}{\rho }\nu$. This implementation modifies Eq. (14) as

$$\underset{f,z}{\arg\;\min}\;\gamma (f)+\psi (z)+\frac{\rho }{2}{\left\|\mathrm{Df}+\mathrm{Bz}-b+w\right\|}_{2}^{2}-\frac{\rho }{2}{\left\|w\right\|}_{2}^{2}.$$ (18)

Then, setting B=−D=−I and b=0, i.e., f=z, Eq. (18) can be split into three optimization problems:

$$f^{\left(k+1\right)}≜\arg\;\underset{f}{\min}\;\gamma (f)+\frac{\rho }{2}{\left\|f-z^{\left(k\right)}+w^{\left(k\right)}\right\|}_{2}^{2},$$ (19)

$$z^{\left(k+1\right)}≜\arg\;\underset{z}{\min}\;\psi (z)+\frac{\rho }{2}{\left\|f^{\left(k+1\right)}-z+w^{\left(k\right)}\right\|}_{2}^{2},$$ (20)

$$w^{\left(k+1\right)}≜w^{\left(k\right)}+\rho (f^{\left(k+1\right)}-z^{\left(k+1\right)}).$$ (21)

The scaled form of ADMM [i.e., Eqs. (19)–(21)] exhibits an improved convergence rate compared with the standard ADMM [i.e., Eqs. (15)–(17)]. The acceleration is achieved by introducing ρ as a scaling factor, which is particularly beneficial for large-scale optimization problems or problems with slow convergence rates. Further insights into these considerations, including heuristics for the effective selection of an appropriate scaling factor, can be found in Ref. 84.

##### Plug-and-play ADMM

The ADMM’s modular structure is one of its main features because it enables the decomposition of a complex optimization problem [i.e., Eqs. (14) or (18)] into several simpler subproblems [i.e., Eqs. (15)–(17) or Eqs. (19)–(21)] that can be solved independently or using established solution methods. Moreover, ADMM’s versatility enables modeling different sparse-based optimization problems. For example, the Tikhonov optimization problem can be modeled by setting $\psi (z)={\left\|z\right\|}_{2}^{2}$ in Eq. (20). As another example, by setting B=Ψ, D=I, b=0, f=Ψz, and $\psi (z)={\left\|z\right\|}_{1}$ in Eq. (18), Eq. (20) can be converted into the basis-pursuit denoising problem. In this regard, a popular framework is the plug-and-play (PnP)-ADMM,^69^ which allows plugging in an off-the-shelf image-denoising algorithm as a solver for the subproblems (see a Matlab implementation in Algorithm 3). In the PnP-ADMM, by setting $\gamma (f)={\left\|\Phi f-g\right\|}_{2}^{2}$, Eq. (19) has the closed-form solution

$$f^{k+1}={\left[\Phi^{T}\Phi +\frac{\rho }{2}I\right]}^{-1}[\Phi^{T}g+\frac{\rho }{2}(z-w)].$$ (22)

Then, Eq. (20) can be rewritten as a denoising problem by setting $\rho =\frac{1}{\sigma^{2}}$, resulting in

$$z^{\left(k+1\right)}=\arg\;\underset{z}{\min}\;\psi (z)+\frac{1}{2\sigma^{2}}{\left\|z-{\overset{˜}{z}}^{\left(k\right)}\right\|}_{2}^{2},$$ (23)

where ${\tilde{z}}^{\left(k\right)}=f^{\left(k+1\right)}+w^{\left(k\right)}$, and σ represents the denoising strength.^88^ Equation (23) can be solved as

$$z=D_{\sigma }({\overset{˜}{z}}^{\left(k\right)})=D_{\sigma }(f^{\left(k+1\right)}+w^{\left(k\right)}),$$ (24)

where $D_{\sigma }$ is a denoiser. Note that the PnP-ADMM algorithm supports any denoiser that fulfills restrictive conditions, such as being non-expansive and having a symmetric Jacobian.^89^ For example, the block-matching and 3D filtering algorithm has been extensively used to enhance the denoising capabilities of the ADMM algorithm while preserving textures and fine details.^90^^,^^91^

**Algorithm 3:** Simulating dual-view CUP’s image reconstruction by a PnP-ADMM algorithm using Matlab^a^.

clear all
close all
clc
%% Load datacube
load(‘Cell.mat’)
[M,N,L] = size(F);
F = F./max(F(:));
n = M*N*L;
mts = M*(N+L-1);
mtu = M*N;
m = mts + mtu;
global m
%% Mask
R = 1*(rand(M,N)>0.5);
R = repmat(R,1,1,L);
%% Sensing matrix
j = 0:n-1;
i = mod(j,M*N)+M*floor(j/(M*N));
Phi_ts = sparse(i+1,j+1,R(:),mts,n);
Phi_tu = kron(ones(1,L),speye(mtu,mtu));
Phi = [Phi_ts;Phi_tu];
%% Measurement
G = Phi*F(:);
G = G/max(G(:));
G = G/L;
%% PnP-ADDM parameters
addpath(genpath(‘./denoisers/RF/’));
dim = size(F);
A = @(F,trans_flag) afun2(F,trans_flag,Phi);
method = ‘RF’;
lambda = 0.25;
opts.rho = 0.1;
opts.gamma = 1;
opts.max_itr = 2000;
opts.print = true;
%% Main routine
F_tilde = PlugPlayADMM_general(F,G,A,lambda,method,opts,dim);

a Functions used in the above script can be downloaded from Ref. 92.

#### 2.2.2 Deep-learning approaches

Deep-learning approaches have been increasingly featured owing to their faster reconstruction compared with their analytical-modeling-based counterparts. Recent advances have allowed embedding mathematical properties offered by the CS theory by designing custom layers that emulate the forward sensing model or exploiting spatiotemporal sparsity via image-denoising nets.^93^ Given access to rich available training datasets, many novel methods based on convolutional neural networks (CNNs) have been developed for CUP’s reconstruction as well as for the encoding mask design, including the end-to-end CNN with residual learning,^94^ the U-Net-based DeepCUP,^95^ the hybrid algorithm that combines the AL method with deep learning,^96^ and the snapshot-to-video autoencoder based on a generative adversarial network.^97^^–^^103^

Here, we review a representative CNN—the deep high-dimensional adaptive net (D-HAN)^104^ that offers multifaceted supervision to CUP by optimizing the encoding mask, sensing the shearing operation, and reconstructing the 3D datacubes. The main goal of the D-HAN is to leverage the merits of both the ADMM and the network-based CS methods by mapping one iteration of the ADMM steps to a deep network architecture. For these reasons, the D-HAN will be used as a benchmark to explain how to link the CUP’s forward model with a CNN approach.

**Fig. 4:**
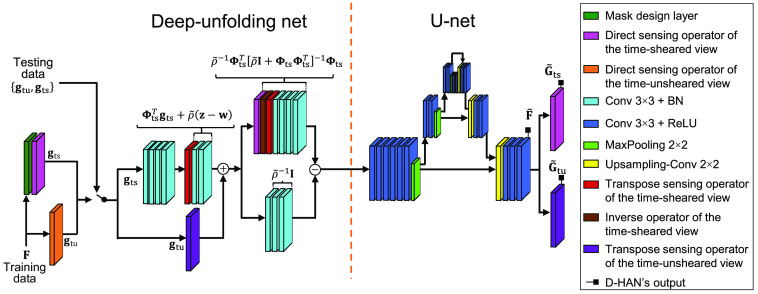
Schematic of the D-HAN for dual-view CUP’s image reconstruction. BN, batch normalization; ReLU, rectified linear activation unit. Adapted with permission from Ref. 104.

Originally designed to use only the time-sheared view, the D-HAN is composed of two cascaded neural networks: a deep-unfolding-based network to embody the sensing model of the time-sheared view in CUP and a U-Net architecture^105^ to further improve image reconstruction (Fig. 4) by exploiting the spatiotemporal correlation of the transient scene. The deep-unfolding net and the U-Net manifest the “divide-and-conquer” approach embedded in the ADMM. Then, the time-unsheared view was incorporated to boost the reconstruction performance by using it as an initialization for the deep-unfolding network and a prior restriction in the loss function. This configuration leverages the original D-HAN’s mathematical advantages and the reduction of unknowns via prior information. This design is memory efficient and thus essential for learning to reconstruct high-dimensional datacubes.

In this regard, the ADMM-based inverse problem can be formulated using the ADMM’s scaled form [i.e., Eqs. (19)–(21)]. Note that in Eq. (19), the analytical inverse model of f refers to a quadratic problem with the closed-form that involves the inversion of a n×n size matrix [see Eq. (22)]. Toward this goal, the Sherman–Woodbury–Morrison (SWM) matrix inversion lemma^106^—a mathematical theorem allowing calculating a matrix’s inverse by converting it into a full rank matrix—and the full-column rank properties are exploited to simplify the process to a smaller-scale matrix inversion and obtain the closed-form solution of the first inverse model in Eq. (22)

$$f={\overset{˜}{\rho }}^{-1}[I-\Phi_{\mathrm{ts}}^{T}{\left[\overset{˜}{\rho }I+\Phi_{\mathrm{ts}}\Phi_{\mathrm{ts}}^{T}\right]}^{-1}\Phi_{\mathrm{ts}}][\Phi_{\mathrm{ts}}^{T}g_{\mathrm{ts}}+\overset{˜}{\rho }(z-w)],$$ (25)

where $\Phi_{\mathrm{ts}}\Phi_{\mathrm{ts}}^{T}\in R^{m_{\mathrm{ts}}\times m_{\mathrm{ts}}}$ represents a matrix product resulting in a diagonal matrix, and $\tilde{\rho }=\rho /2$.

To implement the D-HAN, the first step is to define the operators of dual-view CUP’s data acquisition. First, the direct sensing operators of the time-sheared view and the time-unsheared view, denoted by $G_{\mathrm{ts}}$ and $G_{\mathrm{tu}}$, are expressed as

$$G_{\mathrm{ts}}(F,R)=\overset{L-1}{\underset{l=0}{\sum }}R(\Gamma (l),F_{:,:,l}\circ R),$$ (26)

$$G_{\mathrm{tu}}(F)=\overset{L-1}{\underset{l=0}{\sum }}F_{:,:,l}.$$ (27)

Here, $G_{\mathrm{ts}}(\cdot ):R^{M\times N\times L}\to R^{M\times [N+(L-1)]}$ and $G_{\mathrm{tu}}(\cdot ):\;R^{M\times N\times L}\to R^{M\times N}$. They are shown as the magenta and orange layers in Fig. 4. ∘ represents the Hadamard product. The operator $R(\cdot ):\;R^{M\times N}\to R^{M\times [N+(L-1)]}$ introduces a right-zero-padding (i.e., $\left[F_{:,:,l},0\right]$ with $0\in R^{M\times (L-1)}$) followed by a right-horizontal circular shifting of Γ(l) pixels. A script of Python to construct the sensing operators $G_{\mathrm{ts}}$ and $G_{\mathrm{tu}}$ are presented in Algorithm 4.

**Algorithm 4:** Programming the direct sensing operators of the time-sheared view and the time-unsheared view (i.e., $G_{\mathrm{ts}}$ and $G_{\mathrm{tu}}$) using TensorFlow.

## Direct sensing operator time-sheared view
class DirectSensing_ts(tf.keras.layers.Layer):
def __init__(self, L, M, N, **kwargs):
self.L = L
self.M = M
self.N = N
super(DirectSensing_ts, self).__init__(**kwargs)
def get_config(self):
config = super().get_config().copy()
config.update({
‘bands’: self.L})
return config
def call(self, F, R, **kwargs):
F = tf.multiply(R, F)
F = tf.pad(F, [[0, 0], [0, 0], [0, self.L - 1], [0, 0]],name = “padsensing”)
Gts = None
for i in range(0,self.L):
if Gts is not None:
Gts = Gts + tf.roll(F[:,:,:,i], shift=i, axis=2)
else:
Gts = F[:,:,:,i]
Gts = tf.expand_dims(Gts ,axis=-1)
Gts = tf.math.divide(Gts ,self.L)
return Gts
## → Output: Compressed measurement
## Direct sensing operator time-unsheared view
Gtu = tf.math.reduce_mean(F,axis=-1)

Then, the transpose sensing operators of the time-sheared view and the time-unsheared view, shown as the red and purple layers respectively in Fig. 4, are defined as

$$F_{\mathrm{ts}}(G_{\mathrm{ts}},R)=S(\Gamma (l),G_{\mathrm{ts}}\circ R(\Gamma (l),R)),$$ (28)

$$F_{\mathrm{tu}}(G_{\mathrm{tu}})={\left(G_{\mathrm{tu}}\right)}_{:,:,l}.$$ (29)

Here, $F_{\mathrm{ts}}(\cdot ):\;R^{M\times [N+(L-1)]}\to R^{M\times N\times L}$ and $F_{\mathrm{tu}}(\cdot ):\;R^{M\times N}\to R^{M\times N\times L}$. They return a datacube from a 2D compressed measurement. S(·) is an operator that performs a left-horizontal circular shifting of Γ(l) pixels, followed by the removal of the last (L−1) columns in the resulting shifted matrix to preserve the spatial dimension of the datacube. Algorithm 5 presents a Python script to construct $F_{\mathrm{ts}}$ and $F_{\mathrm{tu}}$.

**Algorithm 5:** Programming the transpose sensing operators of the time-sheared view and the time-unsheared view (i.e., $F_{\mathrm{ts}}$ and $F_{\mathrm{tu}}$) using TensorFlow.

## Transpose sensing operator of the time-sheared view
class Transposesensing_ts(tf.keras.layers.Layer):
def __init__(self, L, M, N, **kwargs):
self.L = L
self.M = M
self.N = N
super(Transposesensing_ts, self).__init__(**kwargs)
def get_config(self):
config = super().get_config().copy()
config.update({
‘bands’: self.L})
return config
def call(self, Gts, R, **kwargs):
F = None
R = R[0,:,:,0]
Gts = Gts[:,:,:,0]
for i in range(0,self.L):
if F is not None:
Ab = tf.roll(Gts, shift=-i, axis=2)
Ax = tf.expand_dims(tf.multiply(R, Ab[:,:,0:self.N]), -1)
F = tf.concat([F, Ax], axis=-1)
else:
Ab = tf.roll(Gts, shift=0, axis=2)
F = tf.expand_dims(tf.multiply(R,Ab[:,:,0:self.N]), -1)
F = self.L*F
return F
## Transpose sensing operator of the time-unsheared view
Gtu = tf.expand_dims(Gtu,axis=-1)
F_tu = tf.broadcast_to(Gtu, [Gtu.shape[0], M, N, L])

Finally, the inverse operator of the time-sheared view, shown as the brown layer in Fig. 4, is defined as

$$I_{\mathrm{ts}}(G_{\mathrm{ts}},R)=G_{\mathrm{ts}}{\circ (\overset{L-1}{\underset{l=0}{\sum }}R(\Gamma (l),R^{{}^{\circ}2})+\overset{˜}{\rho }I)}^{{}^{\circ}-1},$$ (30)

where $I_{\mathrm{ts}}(\cdot ):R^{M\times [N+(L-1)]}\to R^{M\times [N+(L-1)]}$. $(\cdot {)}^{{}^{\circ}2}$ and $(\cdot {)}^{{}^{\circ}-1}$ represent the Hadamard quadratic power and the Hadamard inverse operation, respectively. An example script of Python to construct the inverse operator of the time-sheared view $I_{\mathrm{ts}}$ is summarized in Algorithm 6.

**Algorithm 6:** Programming the inverse operator of the time-sheared view (i.e., $I_{\mathrm{ts}}$) using TensorFlow.

class InverseOperator_ts(tf.keras.layers.Layer):
def __init__(self, L, M, N, **kwargs):
self.L = L
self.M = M
self.N = N
super(InverseOperator_ts, self).__init__(**kwargs)
def get_config(self):
config = super().get_config().copy()
config.update({
‘bands’: self.L})
return config
def build(self, input_shape):
Lambda = tf.constant_initializer(1)
Tau = tf.constant_initializer(1)
Psi = np.zeros([self.M,self.N+self.L-1])
Psi = tf.constant_initializer(Psi)
self.Lambda = self.add_weight(name=“Lbd,” initializer=Lambda, shape=(1),trainable=True)
self.Tau = self.add_weight(name=“Tau,” initializer=Tau, shape=(1),trainable=True,constraint=tf.keras.constraints. MaxNorm(max_value=1, axis=0))
self.Psi = self.add_weight(name=“Psi,” initializer=Psi, shape=(self.M,self.N+self.L-1),trainable=True)
super(InverseOperator_ts, self).build(input_shape)
def call(self, Gts, R, **kwargs):
Gts = Gts[:,:,:,0]
R1 = tf.broadcast_to(R,[1,self.M,self.N,self.L])
Gp = DirectSensing_ts(L=self.L, M=self.M, N=self.N, name=‘DirectPr_InitInv’)(R, R1)
Gp = Gp[:,:,:,0]
Gp = Gp/(self.Lambda) + tf.ones(Gp.shape)
Inv = tf.math.reciprocal(Gp, name=None)
Gts = tf.multiply((self.Tau**2)*Inv+(1-self.Tau**2)*self.Psi,Gts)
Gts = tf.expand_dims(Gts,axis=-1)
F = TransposeSensing_ts(L=self.L, M=self.M,N=self.N, name=‘TransPr_InitInv’)(Gts,R)
#
F = F/(self.Lambda**2)
return F

Following the definition of these five operators, the next step is to model the SWM matrix approach. Toward this goal, Eq. (25) is split into two main equations $\Phi_{\mathrm{ts}}^{T}g_{\mathrm{ts}}+\overset{˜}{\rho }(z-w)$ and ${\overset{˜}{\rho }}^{-1}I-{\overset{˜}{\rho }}^{-1}\Phi_{\mathrm{ts}}^{T}{\left[\overset{˜}{\rho }I+\Phi_{\mathrm{ts}}\Phi_{\mathrm{ts}}^{T}\right]}^{-1}\Phi_{\mathrm{ts}}$. In the D-HAN, the first equation is reflected as $F_{\mathrm{ts}}$ coupled to two 2D convolutional layers, each of which with a batch normalization operation (referred to hereafter as a 2D convolutional + batch normalization (BN) layer and shown in cyan in Fig. 4). The output from the 2D convolutional + BN layer is added with an estimate from the time-unsheared view generated by $G_{\mathrm{tu}}$ and $F_{\mathrm{tu}}$. Subsequently, the second equation is represented by two parallel arms. The upper arm, corresponding to ${\overset{˜}{\rho }}^{-1}\Phi_{\mathrm{ts}}^{T}{\left[\overset{˜}{\rho }I+\Phi_{\mathrm{ts}}\Phi_{\mathrm{ts}}^{T}\right]}^{-1}\Phi_{\mathrm{ts}}$, is composed of $G_{\mathrm{tu}}$ as a first layer followed by $I_{\mathrm{ts}}$ and $F_{\mathrm{ts}}$ along with four 2D convolutional + BN layers. The bottom arm, which corresponds to ${\tilde{\rho }}^{-1}I$, has three 2D convolutional + BN layers. The outputs of both arms are subtracted and given as the input to the U-Net in the D-HAN that reflects Eq. (24). In the U-Net, the datacube passes through an encoding pathway comprised of max-pooling layers that simultaneously reduce the spatial dimension and increase the channels. This down step returns a smaller-size datacube with the more meaningful high-level details of the image (e.g., edges, textures, or shapes) linked to the scene’s sparsity. Then, in the decoding step, comprised of upsampling layers, the U-Net reconstructs the full-size datacube (denoted by $\overset{˜}{F}$) using these learned high-level details.

The loss function L(·), used to learn the D-HAN’s weights, is established as

$$L(F)=l_{1}(F,\overset{˜}{F})+l_{1}(G_{\mathrm{ts}},{\overset{˜}{G}}_{\mathrm{ts}})+l_{1}(G_{\mathrm{tu}},{\overset{˜}{G}}_{\mathrm{tu}})+l_{\mathrm{SSIM}}(F,\overset{˜}{F}),$$ (31)

where $\overset{˜}{F}$ is the D-HAN’s output, ${\overset{˜}{G}}_{\mathrm{ts}}$ and ${\overset{˜}{G}}_{\mathrm{tu}}$ are estimations of the compressed measurement from $\overset{˜}{F}$ using Eqs. (26) and (27), respectively. $l_{1}(\cdot )$ is the $l_{1}$-norm operator, and $l_{\mathrm{SSIM}}(\cdot )$ represents the structural similarity (SSIM) index.^107^

#### 2.2.3 Simulation

CUP’s image reconstruction of the “cell-division” scene is simulated using both the analytical-modeling-based algorithm (in Matlab) and deep-learning algorithm (in Python). The dimensions of the datacube were set as M×N×L=256×256×25  pixels, and the binary mask holds the structure proposed in Ref. 104. Four popular databases—“SumMe,”^108^ “Need for Speed,”^109^ “Sports Videos in the Wild,”^110^ and “Mouse Embryo Tracking”^67^—were used to train the D-HAN. The PnP-ADMM algorithm and a pretrained version of the D-HAN can be downloaded from Ref. 92 (Matlab 2022b) and Ref. 111 (Python, TensorFlow). In addition, a more beginner-friendly Python version is available in Ref. 112, which was trained on the Google Colaboratory (CoLab) application—a free Jupyter Notebook interactive development environment for Python hosted in Google’s cloud.

**Fig. 5:**
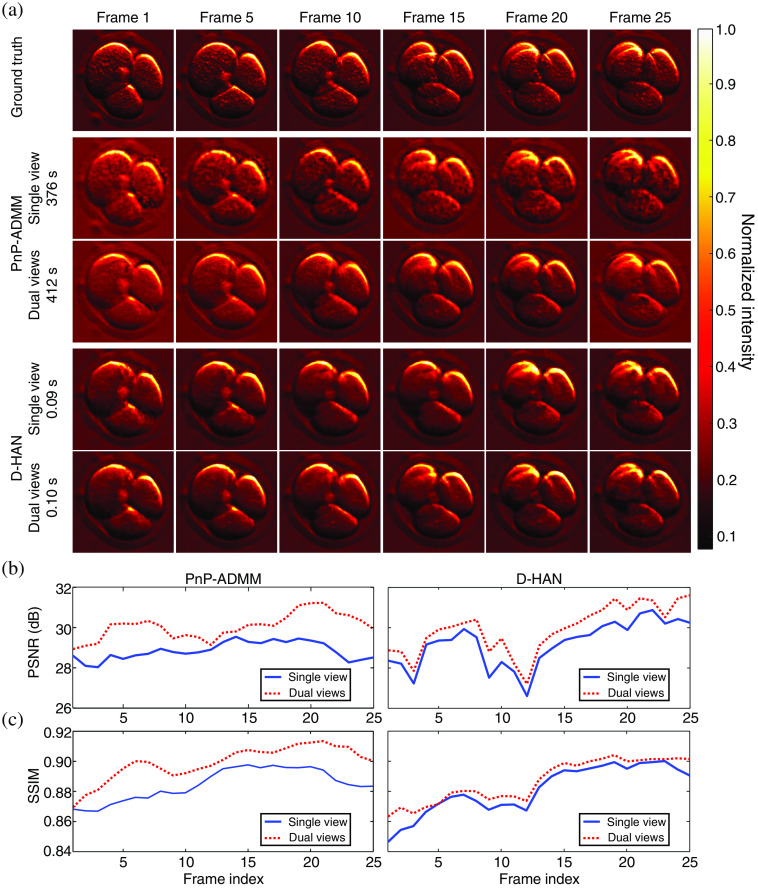
Simulation of CUP’s image reconstruction of the “cell-division” dynamic scene using the PnP-ADMM algorithm and the D-HAN. (a) Six selected frames of the ground truth and the single-view and dual-view reconstructions using the PnP-ADMM and D-HAN algorithms. (b) PSNR of each reconstructed frame. (c) As (b), but showing the SSIM index (Video 1, MP4, 408 KB [URL: https://doi.org/10.1117/1.JBO.29.S1.S11524.s1]).

Six exemplary frames of the scene (as the ground truth) and their corresponding frames reconstructed by single-view and dual-view CUP using the PnP-ADMM and the D-HAN are shown in Fig. 5(a). The movie is shown in Video 1. As shown in Figs. 5(b) and 5(c), results show that implementing the dual-view approach exceeds the reconstruction performance of a single-view CUP in terms of the average peak signal-to-noise ratio (PSNR) defined as PSNR¯=1L∑l=0L−1[10 log10([max(F:,:,l)]2mtu−1‖vec(F:,:,l)−vec(F^:,:,l)‖22)] and the average SSIM index^113^ defined as SSIM¯=1L∑l=0L−1[[Lum(F:,:,l,F^:,:,l)]α[Cont(F:,:,l,F^:,:,l)]β[Struc(F:,:,l,F^:,:,l)]γ]. Here, vec(·) is a vectorization operator, and F^ is the reconstructed result. The operators $\mathrm{Lum}(\cdot )=\frac{2\mu_{x}\cdot \mu_{y}+C_{1}}{\mu_{x}^{2}+\mu_{y}^{2}+C_{1}}$, $\mathrm{Cont}(\cdot )=\frac{2\sigma_{x}\cdot \sigma_{y}+C_{2}}{\sigma_{x}^{2}+\sigma_{y}^{2}+C_{2}}$, and $\mathrm{Struc}(\cdot )=\frac{\sigma_{xy}+C_{3}}{\sigma_{x}\sigma_{y}+C_{3}}$ measure the similarities in luminance, contrast, and structure, respectively, where $\mu_{x},\mu_{y},\sigma_{x},\sigma_{y}$, and $\sigma_{xy}$ are the local means, standard deviation, and cross-covariance for the images. {α,β,γ}>0 are parameters used to adjust the relative importance of the three components. $C_{1},C_{2}$, and $C_{3}$ are constants to stabilize the division with weak denominator. For the results shown in Fig. 5, SSIM’s parameters were set as α=β=γ=1, $C_{1}=0.01^{2}$, $C_{2}=0.03^{2}$, and $C_{3}=C_{2}/2$. The D-HAN obtains a better average PSNR and a comparable average SSIM to the PnP-ADMM approach in both single-view and dual-view CUP.

CUP’s performance decreases with higher noise and stronger compression. Table 1 illustrates this general trend from an ablation analysis of the “cell-division” dynamic scene using the PnP-ADMM algorithm. Higher noise levels reduce spatial resolution. Higher compression ratios result in stronger blurring in the temporal shearing direction, which further decreases the spatial resolution in that direction.^59^^,^^91^^,^^114^ Both factors hamper the reconstruction algorithm’s ability to accurately place the correct amount of intensity from the compressed snapshot to the appropriate spatiotemporal position in the reconstructed datacube.

**Table 1:** Average PSNRs in reconstructed datacubes with different compression ratios and signal-to-noise ratios (SNRs).

Compression ratio	SNR (dB)				
	15	20	25	30	Infinity
10.1×	26.3 ± 0.8	27.9 ± 0.5	29.5 ± 0.7	30.0 ± 0.8	30.4 ± 0.9
11.9×	26.2 ± 0.6	27.7 ± 0.5	29.2 ± 0.6	29.9 ± 0.7	30.2 ± 0.8
16.4×	26.1 ± 0.5	27.5 ± 0.5	28.7 ± 0.5	29.7 ± 0.7	29.9 ± 0.7
22.9×	25.8 ± 0.8	27.4 ± 0.4	28.2 ± 0.6	28.8 ± 0.7	28.9 ± 0.8
45.6×	25.6 ± 0.9	27.1 ± 0.8	28.1 ± 0.9	28.6 ± 1.0	28.8 ± 1.1

## 3 System

The construction of a CUP system involves a careful selection of three crucial components. First, a spatial encoder modulates the dynamic event. Second, a temporal shearing unit deflects the spatially encoded frames to different spatial positions according to their time of arrival. Finally, a 2D sensor integrates the spatially encoded and temporally sheared datacube into the time-sheared view. For dual-view CUP, another 2D sensor integrates the dynamic scene into the time-unsheared view. To date, many approaches have been implemented to devise each component. A comprehensive survey of these implementations, their advantages, and limitations will be presented in this section, followed by a discussion on important steps to calibrate a CUP system.

### 3.1 Spatial Encoder

**Fig. 6:**
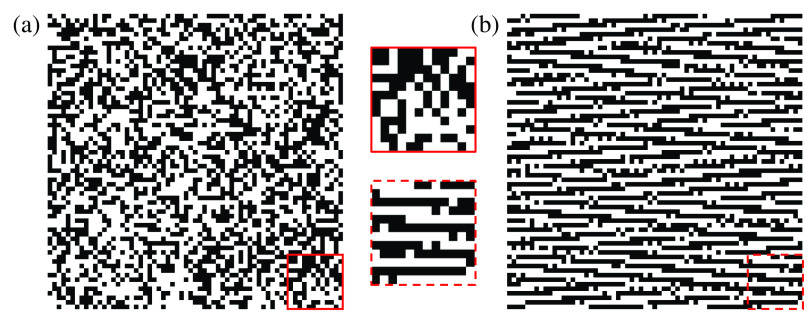
Representative encoding patterns for CUP. (a) Pseudorandom pattern. (b) Deep-learning-optimized pattern. Insets: Zoomed-in views of local regions.

The selection of a suitable spatial encoder in a CUP system includes encoding pattern design and the encoder’s implementation. Because CUP relies on CS principles, its sensing matrix can be designed based on the restricted isometry property (RIP) to ensure its incoherence to the representation matrix of the scene. Notably, the sensing matrix based on a random pattern has been verified to meet the RIP criterion for a wide range of representation bases.^115^ Therefore, pseudorandom masks [Fig. 6(a)] are dominantly implemented as spatial encoders in reported CUP systems.

The RIP also provides a valuable metric for evaluating the encoder’s quality. The general strategy is to reduce the coherence between the sensing matrix and the representation matrices to ensure that the projection of high-dimensional data onto a lower-dimensional space preserves the essential data features.^116^^,^^117^ It guarantees that the compressed measurements retain sufficient information to accurately reconstruct the original signal. To date, several works have improved the mask via deep learning.^104^^,^^118^^,^^119^ As an example, an encoding mask designed via the D-HAN is shown in Fig. 6(b), where the shearing operation in CUP’s forward model and the training data produce horizontal stripe-like structures.^104^

**Fig. 7:**
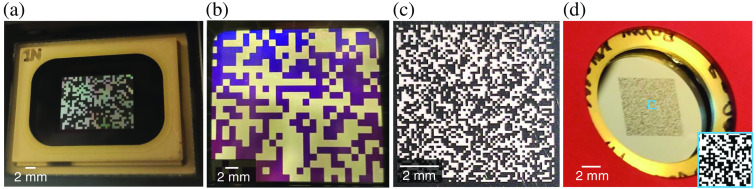
Pseudorandom binary masks displayed on representative spatial encoders. (a) DMD. (b) LC-SLM. (c) Plastic mask fabricated by high-definition printing. (d) Chromium mask made by photolithography. Inset: Zoom-in view of a local region.

As summarized in Table 2, four approaches have been used to implement CUP’s spatial encoders: digital micromirror devices (DMDs),^17^^,^^66^^,^^102^^,^^120^^,^^121^ liquid-crystal spatial light modulators (LC-SLMs),^134^ high-definition printing,^97^^,^^135^ and photolithography.^91^ Among them, the DMD, as a reflective binary-amplitude spatial light modulator,^136^ can provide reconfigurable, stable, and broadband encoding [Fig. 7(a)]. However, due to the micromirror’s tilt angle, the DMD is often required to be placed in the Littrow configuration in CUP systems^17^^,^^37^^,^^59^ to retro-reflect the incident light. Since the DMD is not parallel to the object plane, this design limits the field of view (FOV). Moreover, the DMD’s structure limits light efficiency in three main aspects. First, since the DMD has a ∼94% fill factor, a part of incident light is lost in the gaps between neighboring micromirrors. Second, as a 2D diffraction grating,^136^ the DMD has an overall diffraction efficiency of ∼86%,^137^ which indicates energy loss in high-diffraction orders. Finally, the aluminum coating of the micromirrors has a reflectivity of 89% in the visible spectrum with a dip at around 800 nm corresponding to the absorption of inter-band transitions in aluminum. Consequently, the constructed CUP system may not have an optimal spectral response for the dynamic scenes under investigation.

**Table 2:** Representative approaches for CUP’s spatial encoders.

Approach	Advantage	Limitation	References
DMD	— Programmable encoding	— Restricted FOV due to the Littrow configuration	17, 55, 57, 58, 60–62, 64–66, 96, 99, 102, 104, 114, 116, 120–133
	— Broad operating spectrum	— Energy loss due to the limited fill factor and diffraction	
		— Nonoptimal spectral response to the micromirror’s coating	
LC-SLM	— Programmable encoding	— Wavelength and polarization sensitive modulation	134
	— Phase and amplitude modulation in grayscale	— Relatively low fill factor for the transmissive type	
	— Reflective and transmissive encoding ability	— Flicker noise	
High-definition printing	— Transmissive encoding	— Unreconfigurable encoding	97, 135
	— Low cost		
	— Broad operating spectrum		
Photolithography	— Transmissive encoding	— Unreconfigurable encoding	91
	— High resolution		
	— Broad operating spectrum	— High cost	

Another choice of reconfigurable spatial encoding is LC-SLMs. They have been widely implemented in coded optical imaging.^18^^,^^138^^–^^141^ LC-SLMs can simultaneously modulate amplitude and phase in grayscale.^134^ In the context of CUP, they can provide both reflective and transmissive spatial encoding [Fig. 7(b)]. Nonetheless, LC-SLMs could also bring in some limitations in spatial encoding. Its modulation is sensitive to both the wavelength and polarization. Moreover, a relatively low fill factor of transmissive LC-SLMs (e.g., 58%)^142^ and the flicker noise could limit pattern quality and encoding stability.^143^

Besides using the programmable devices, an encoded mask can be directly fabricated on a substrate. As a representative approach, high-definition printing can manufacture encoding masks at up to 50,800 dots per in. resolutions, with up to ∼30  in.×30  in. in size, at $16.7 per ${\mathrm{in.}}^{2}$ (Ref. 144) [Fig. 7(c)]. In one printing task, users can pack multiple masks with different encoding pixel sizes down to 7  μm and different pattern types as well as calibration patterns, such as single pinholes, pinhole arrays, and slits. As another approach, photolithography can produce spatial encoders with nanometer-level encoding pixel sizes over inches [Fig. 7(d)]. As an example, a 3-in. × 3-in. mask with 125-nm resolution can be fabricated at ∼$6,000. As a well-established fabrication technique, photolithography can be used with various materials to target different spectral bands.^145^ These fabricated coded masks can be directly inserted in CUP systems, which conserves space for a more compact system design. Although capable of providing broadband and transmissive encoding, these two approaches can only prepare fixed spatial encoders. In addition, the almost unavoidable defect pixels in the fabricated encoder request careful calibration to build an accurate sensing matrix.

### 3.2 Temporal Shearing Unit

Depending on the necessity of external power, temporal shearing units can be classified into passive units and active units (Table 3). The former deflects the temporal information transferred to certain photon tags (e.g., wavelengths) by exploiting the properties in these tags (e.g., color dispersion). Being jitter-free, these compact units bring in stable operation without increasing the control complexity.^59^ The active units are driven by time-varying electric signals to trigger deflection. Usually integrated into the detection side of the imaging systems, they enable receive-only detection, which is specifically suited for capturing self-luminescent and color-selective events.^16^^,^^18^^,^^66^

**Table 3:** Representative methods for CUP’s temporal shearing units.

Category	Approach	Advantage	Limitation	References
Passive	Grating	— Compact	— Requirement of chirped pulse illumination	29, 59, 121
		— Low cost		
		— Jitter-free	— Fixed shearing rate	
		— Ultrafast shearing		
	Metalens	— Compact, lightweight, and less complex optomechanically	— Requirement of chirped pulse illumination	146
		— Joint temporal shearing and imaging	— Fixed shearing rate	
		— Jitter-free	— Limited aperture size	
		— Ultrafast shearing	— High cost	
Active	Image-converter streak tube	— Receive-only detection	— High cost	17, 55, 57, 58, 60–62, 64–66, 91, 96, 99, 102, 104, 114, 116, 120, 121, 123–131, 147
		— Tunable shearing speeds	— Space-charge effect	
		— Ultrafast shearing	— Electronic jitter	
			— Low overall efficiency	
			— Spectra limited by the photocathode	
	Rotating mirror	— Receive-only detection — Tunable shearing speeds	— Relative slow shearing speed	37, 97, 148, 149
		— All-optical operation		
		— Broad operating spectrum		
		— Low cost		
	TDI technique	— Receive-only detection	— Fixed shearing speed	134, 150
		— Joint temporal shearing and spatiotemporal integration	— Relative slow shearing speed	
	Electro-optical deflector	— Receive-only detection	— Small numerical aperture	122
		— All-optical operation	— High operating voltage	
		— Ultrafast shearing	— Limited deflection angle	
	Molecular deflector	— Receive-only detection	— Requirement of an ultrafast, high-intensity pump laser pulse	151
		— All-optical operation		
		— Small size		
		— Ultrafast shearing		

**Fig. 8:**
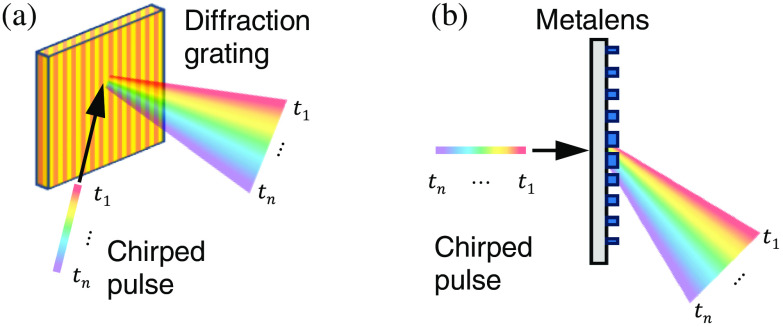
Representative passive temporal shearing units for CUP. The temporal information is mapped to the spectrum and deflected to different spatial positions by (a) a diffraction grating and (b) a metalens. $t_{1}$ to $t_{n}$, temporal information.

Figure 8 shows two examples of passive temporal shearing units. Both need to team up with a chirped ultrashort probe pulse, which maps the temporal information of the event to its spectral band. As shown in Fig. 8(a), the modulated chirped pulse is spatially dispersed by a grating.^59^ In recent years, the development of metamaterials has made metalenses a potential passive temporal shearing unit. They consist of an array of waveguide structures with a subwavelength size, with resonant metamaterial elements etched into the surface [Fig. 8(b)].^152^^,^^153^ Metalenses can strongly disperse light while manipulating its phase, amplitude, and polarization.^154^ This property has been exploited in hyperspectral imaging.^155^ Grafting this sensing paradigm in CUP, a metalens integrates imaging and temporal shearing, which greatly reduces the system’s size and complexity.^146^ Besides the aforementioned two units, other dispersive optical elements such as kinoforms,^156^ zone plates,^157^ and diffractive optical elements (DOEs)^158^ could also be used for passive temporal shearing of chirped pulses.

**Fig. 9:**
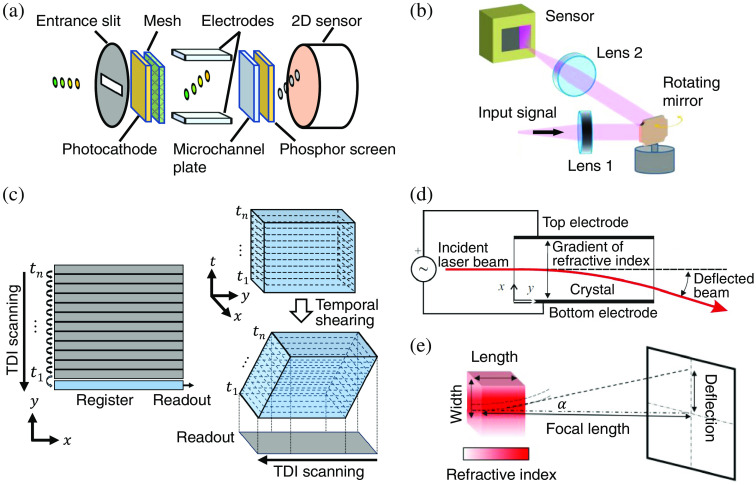
Representative active temporal shearing units for CUP. (a) Image-converter streak tube. (b) Rotating mirror. (c) TDI mode of a CCD camera. (d) Electro-optical deflector. (e) Ultrashort-pulse-induced ${\mathrm{CO}}_{2}$ molecule deflector. α, Deflection angle. (b) Reprinted with permission from Ref. 135. (d) Adapted with permission from Ref. 159. (e) Adapted with permission from Ref. 151.

Active temporal shearing units have also been featured in many CUP systems. As an example, the image-converter streak tube is shown in Fig. 9(a). Such a device works by directing the dynamic scene onto a photocathode, where the incident photons are converted to photoelectrons. After being accelerated by a pulling voltage added on a metal mesh, these photoelectrons are temporally sheared by a varying electric field produced by applying a voltage to a pair of sweep electrodes. Then, the photoelectric signal is amplified by a microchannel plate. Finally, the photoelectrons bombard a phosphor screen and are converted back to photons.^59^^,^^91^ The configuration of the image-converter streak tube takes advantage of the movement of electrons under high-voltage electric fields, enabling ultrafast shearing for the CUP system to provide up to femtosecond-level temporal resolution.^58^^,^^59^ However, this operation is inevitably affected by the electronic jitter. Moreover, due to the space-charge effect in electronic imaging,^160^ a trade-off needs to be made between the incident light intensity and the signal gain, which limits the imaging quality of the streak tube-based CUP systems.^37^^,^^151^ The efficiency of image-converter streak tubes is also inherently limited by the photon–electron–photon conversion. The quantum yield of the photocathode is moderate for the visible light and decreases dramatically for near-infrared light.^161^^,^^162^ The phosphor screen also has a relatively low conversion efficiency, especially for the fast-responding types.^163^ The limited overall efficiency makes the image-converter streak tubes less suitable for imaging faint transient events.

Rotating mirrors are another popular choice of active temporal shearing units for CUP. The mirror rotation continuously alters the angle of incidence, hence shearing the reflected light [Fig. 9(b)]. Rotating mirrors are preferred to be placed at the Fourier plane of a 4f-system so that after the second lens, the chief rays of all temporal frames can propagate and enter the sensor perpendicularly, which avoids aberrations introduced by the field curvature.^164^ Producing tunable temporal resolutions typically from hundreds of nanoseconds to microseconds, they are much slower than the image-converter streak tube. However, the all-optical operation avoids the space-charge effect, which enables optics-limited spatial resolution and high dynamic ranges.^37^ Moreover, by circumventing the photon-to-photoelectron conversion in a photocathode, rotating-mirror-based CUP systems can employ sensors in matching responsive bands to sense photons with relatively low energy (e.g., in the infrared range). Leveraging high reflectivity coatings (e.g., >95% at 0.4 to 20.0  μm),^165^ these CUP systems are attractive candidates for high-sensitivity transient imaging at broad spectral bands.

Besides these two popular approaches, other specialized optical and/or electronic devices have been implemented as CUP’s temporal shearing units. As an example, Fig. 9(c) shows the operation principle of the time-delay-integration (TDI) mode of a CCD camera. Initially developed to visualize moving objects under extremely low light levels, the TDI configuration employs a long exposure during which the generated photoelectrons shift down row by row before eventually reading out.^166^ In this way, the read-out data are the integration of information from different rows at different time points. Such a mechanism enables TDI cameras to combine the operations of temporal shearing and spatiotemporal integration, which considerably reduces the system’s complexity.^134^^,^^150^ Recently, electron-transfer-based temporal shearing has also been implemented in a streak-camera sensor.^167^^,^^168^ Hundreds of sampling and storage cells are placed underneath a line of photodiodes. During the sensor’s exposure, the 1D signal is sampled and sequentially stored at a temporal resolution of 500 ps. Although its 1D FOV excludes its implementation with CUP, this highly integrated device marks its potential to be further developed for future CUP systems.

Electro-optic crystals can also be used as the temporal shearing unit of CUP systems. As shown in Fig. 9(d), a time-varying electric field is applied to modulate the gradient of the refractive index of an electro-optic crystal. In this way, this electro-optic deflector (EOD) can direct the incident light to different propagation directions according to its time of arrival.^159^^,^^169^ The EOD is currently the only all-optical shearing unit capable of achieving $50\times 10^{9}$ frames per second (fps) in a CUP system.^122^ However, the shortcomings of small numerical aperture, high operating voltage, and limited deflection angle still hinder EODs for further applications in CUP.

Finally, transient materials’ behaviors have been proposed as CUP’s temporal deflectors. Figure 9(e) depicts how the transient alignment of ${\mathrm{CO}}_{2}$ molecules excited by an ultrashort laser pulse can induce a time-varying refractive-index gradient, resulting in different deflection angles to temporally shear the dynamic scene.^151^ Although having not been experimentally demonstrated, this mechanism could open a new avenue of transient-event-assisted ultrafast imaging. The fast responses of properly selected materials could push CUP’s imaging speed to the quadrillion fps level.^170^

### 3.3 2D Sensor

After being spatially encoded and temporally sheared, the dynamic scene is spatiotemporally integrated over each pixel by a 2D sensor. Most of the current commercial cameras (e.g., CCD, CMOS, scientific CMOS, and electron-multiplying CCD cameras) have been implemented to construct a CUP system. Nonetheless, used as the last component in a CUP system, the 2D sensor needs to be carefully selected to accommodate the characteristics of the dynamic scenes, the spatial encoders, and the temporal shearing units. In terms of spectral responsiveness, the 2D sensors are desired to have the highest sensitivity at the corresponding spectra of the dynamic scene. However, it might be restricted by the device. For example, for the image-converter streak tube, the quantum yield of the deployed camera should be peaked at the wavelength of the phosphor screen (e.g., 540 nm of a P43 phosphor screen). The pixel size of these sensors is required to sufficiently sample each encoding pixel for the given system’s magnification.

**Fig. 10:**
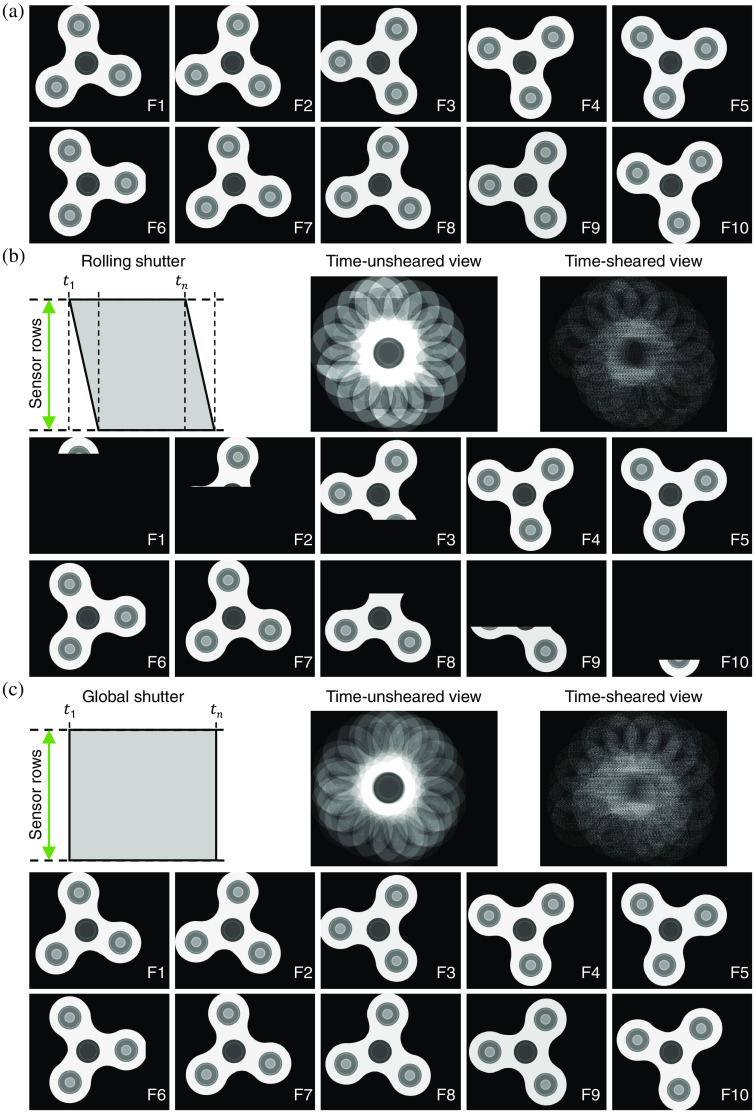
Comparison between the rolling shutter and the global shutter in CUP’s data acquisition. (a) 10 representative frames of a simulated rotating-spinner scene. (b) Rolling shutter’s operating principle (top-left panel), the produced 2D snapshots (top-middle and top-right panels), and the illustration of CUP’s reconstructed frames (bottom panel). (c) As (b), but showing the results produced by the global shutter. F1 to F10, frame indices.

The shutter type is another important factor in the sensor selection. Overall, the global shutter is much preferred for CUP operation compared with the rolling shutter. Figure 10(a) shows a simulated dynamic scene of a rotating spinner with constant intensity. For a rolling-shutter sensor, the exposure of each row starts sequentially from the top to the bottom for the same period and ends at a different time point. The induced rolling-shutter effect distorts the image of fast-moving objects.^171^ CUP can overcome this distortion by putting the information back in the correct spatiotemporal position. However, due to the different starting times of exposure, only a part of FOV can be reconstructed for images at the beginning and the end of the movie, as shown in Fig. 10(b). This issue can be bypassed by limiting the occurrence of the dynamic event when all rows are under exposure. In contrast, the global shutter, which can be implemented in both CCD and CMOS sensors, allows capturing the dynamic scene over the full FOV [Fig. 10(c)] and thus avoid the time-windowing effect of the rolling shutter.

### 3.4 Calibrations

In this section, we outline a few important calibration steps in CUP’s operation. They are necessary for both physical data acquisition and computational reconstruction of the dynamic scene.

#### 3.4.1 Co-registration of multiple views

**Fig. 11:**
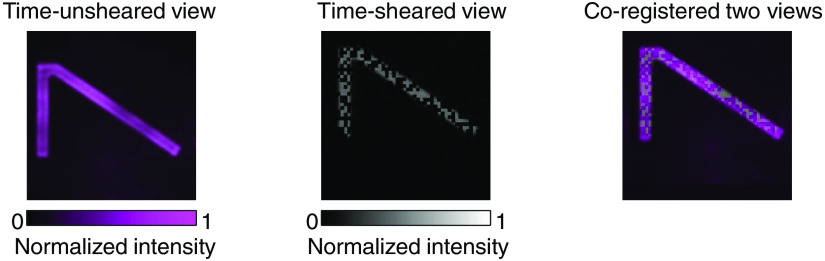
Co-registration for dual-view CUP.

Due to the difference among individual imaging arms, the acquired snapshots may have different aberrations. It is thus indispensable to accurately co-register all the views for accurate image reconstruction. Toward this goal, a static image of the time-sheared view is acquired by turning off the shearing unit (Fig. 11). In Matlab, the co-registration can be carried out using “control point registration” in the “Image Processing Toolbox.”^172^ The function “cpselect” opens a window for the user to select at least four pairs of control points in both views. Then, the function “fitgeotform2d” estimates the transformation matrix that best aligns the control points. Finally, the function “imwarp” applies the transformation matrix to complete the co-registration. The co-registered time-unsheared view and the time-sheared view are then fed into the reconstruction algorithms.

#### 3.4.2 Acquisition of the encoding mask

**Fig. 12:**
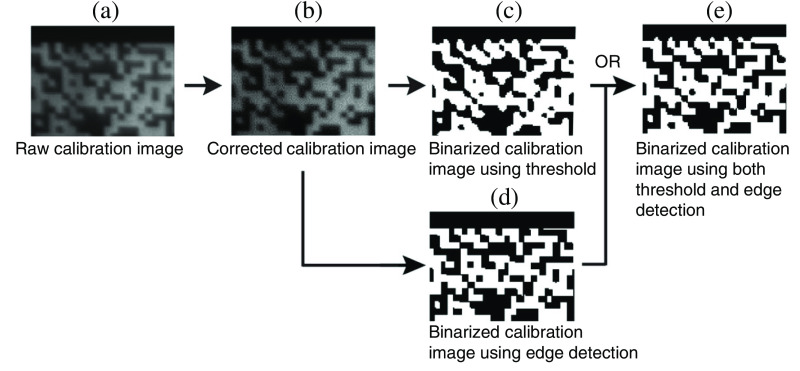
Binarization of the captured encoding mask image. (a) Section cropped from the acquired mask image. (b) Cropped section after background subtraction and white-field correction. (c) Image binarization by applying a threshold to (b). (d) Image binarization by detecting edges in panel (b). (e) Combining (c) and (d) using OR operation. Adapted with permission from Ref. 57.

The experimentally captured encoding mask image produces better reconstruction than the design file of the used pattern because it takes into consideration various practical imperfections. For example, the DMD’s micromirrors may have a different orientation than those of the sensors. The fixed encoders fabricated by high-definition printing or photolithography also have defective pixels or membrane curving. These cases cannot be eliminated even if the imaging system is tuned with a proper magnification that matches the size of the encoding pixels to that of the sensor’s pixels. Thus, a mask image is captured by tuning off the shearing unit and then binarized for CUP’s image reconstruction (Fig. 12). Besides background subtraction and white-field correction, threshold selection and edge detection are combined to optimize binarization.^57^ This calibration can also reduce aberrations and field curvature.^123^ In practice, the FOV and the maximum shearing distance are also limited to ensure high quality in captured images.

#### 3.4.3 Linearity test of shearing operation

**Fig. 13:**
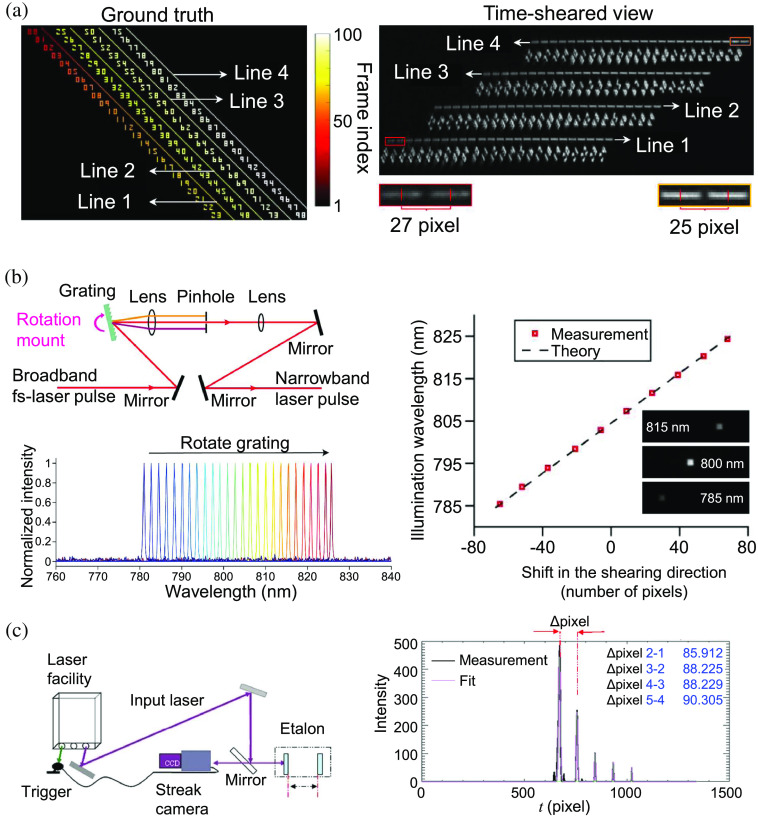
Linearity test of the temporal shearing operation. (a) Test of a rotating-mirror-based temporal shearing. Left panel: composite of the 100 frames with consecutive short lines and frame indices. Right panel: analysis of displacement in the time-sheared view. (b) Test of a diffraction-grating-based temporal shearing. Left panel: setup (top) of generating pulses with selected wavelengths (bottom). Right panel: result of the linearity test by illuminating a square pattern with the narrow-band pulses. (c) Test of a streak-tube-based temporal shearing. Left panel: setup of the test. Right panel: cross-section in the streak measurement of the pulse train generated by the etalon. (a) Adapted with permission from Ref. 104. (b) Adapted with permission from Ref. 59. (c) Adapted with permission from Ref. 174.

Linear temporal shearing is used in CUP’s forward model (see Sec. 2.1). However, various experimental factors could deviate the linear temporal shearing operation, including misalignment, jitter, and imperfect instrument responses.^173^ Therefore, a linearity test is required to assess the system’s performance and to compensate for these factors. An example of a rotating-mirror-based CUP system is shown in Fig. 13(a). About 100 frames containing number indices and short lines were displayed on a DMD at 20 kHz. From the recorded snapshot, the displacements between the centroids of the consecutive short lines were calculated to determine the rotating mirror’s shearing operation. In this example, the shearing deviates from a linear function by 2 pixels over 100 frames.^104^

As another example, Fig. 13(b) shows the linearity test of a diffraction-grating-based CUP system.^59^ A tunable bandpass filter was built based on a rotating grating [top-left panel in Fig. 13(b)] to produce pulses with a selected wavelength [bottom-left panel in Fig. 13(b)]. The generated narrowband pulses illuminated a small square pattern, whose positions in the streak images were measured to obtain the relationship with wavelengths [right panel in Fig. 13(b)]. Finally, an example of the linearity test of an image-converter streak tube is shown in Fig. 13(c). Following a calibration protocol similar to that of the diffraction grating-based CUP system, a pulse train with a known interval was generated by an etalon. The linearity was computed by measuring the deflected pulses’ positions.^174^

## 4 Biomedical Applications

Many biological processes, such as blood flow, brain activities, or cellular dynamics, are not repeatable. Single-shot CUP provides an innovative and complementary tool to probe these events, which generates valuable insights for the fundamental understanding of their underlying mechanisms. In this section, we will focus on four representative biomedical applications of CUP.

### 4.1 Neuroimaging

Monitoring the spatiotemporal dynamics of neuron signaling is essential to the understanding of the brain’s structure and function. Direct visualization can aid researchers and clinicians in studying neurological disorders, cognitive processes, and brain development. Frame rates at the level of one billion fps are demanded to image the propagation of action potentials (APs) in myelinated axons (∼100  m/s) with high spatial resolution and in real time. Unreachable by conventional electronic sensors, this requirement poses a considerable technical challenge to neuroimaging research.

**Fig. 14:**
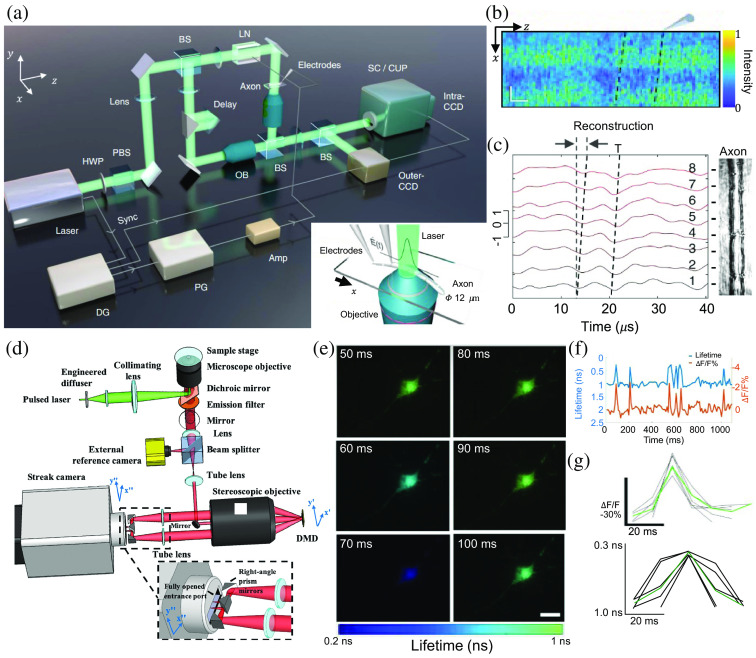
CUP of neuronal activities. (a) Schematic of Diff-CUP. Inset: Adhesion microscope slide for Diff-CUP imaging. BS, beam splitter; DG, delay generator; E(t), transient field stimulation; HWP, half-wave plate; LN, lithium niobate; OB, objective lens; PBS, polarizing beam splitter; PG, pulse generator; SC, streak camera. (b) Spatiotemporal interferogram of a propagating internodal current flow in a myelinated axon captured by Diff-CUP. (c) Reconstruction of the current flow signals based on the stimulus interferogram. Black dashed lines indicate the signal region of the internodal current flow. T, the propagation time of the internodal current flow within the FOV. (d) Schematic of compressed FLIM. (e) Six representative frames from the reconstruction of a cultured hippocampal neuron upon potassium stimulation at 100 fps. (f) Time-lapsed lifetime and intensity curves of a cultured hippocampal neuron. (g) Intensity (top panel) and lifetime (bottom panel) waveforms of neural spikes (black lines) and their means (green lines) for a cultured hippocampal neuron under stimulation. (a)–(c) Adapted with permission from Ref. 65. (d)–(g) Adapted with permission from Ref. 60.

Overcoming this challenge, CUP imaged phase and lifetime dynamics evoked by neuronal activities. As an example, by combining Mach–Zehnder interferometry and utilizing its ultrafast imaging speed and large sequence depth, differentially enhanced CUP (Diff-CUP) imaged internodal current flow in myelinated axons from the sciatic nerves of *Xenopus laevis* frog at $200\times 10^{9}\;\mathrm{fps}$^65^ [Fig. 14(a)]. The high phase sensitivity of Diff-CUP enables simultaneously capturing the substantial cellular deformations and consequent phase alterations induced by passive current flows (i.e., without the amplification of the electrical current)^175^^,^^176^ resulting from a 10-V and 1-μs pulse injected into the axon [Fig. 14(b)]. The reconstructed correlation curves of each segment of the FOV (labeled with numbers 1 to 8) reveal the microsecond-level phase changes induced by the propagating internodal current flow [Fig. 14(c)], whose conduction speeds in myelinated axons were calculated to be 100±26  m/s. To date, Diff-CUP is the fastest imaging-based approach for assessing AP-related conduction.

CUP is also implemented as a CS-based fluorescence lifetime imaging microscopy (FLIM)^60^ to record high-resolution 2D lifetime images of immunofluorescently stained neurons [Fig. 14(d)]. With an imaging speed of $∼10\times 10^{9}\;\mathrm{fps}$, this CUP-based FLIM system captured the fluorescence intensity decay in real time, which produced a 2D lifetime map. Leveraging the intrinsic frame rate of the internal CMOS camera, lifetime maps were generated at 100 fps. This technique visualized neural spike dynamics via the fluorescence intensity and donor lifetime decrease during Förster resonance energy transfer.^177^ Figure 14(e) illustrates six representative lifetime images of a cultured hippocampal neuron at 100 fps. The time courses of the averaged fluorescence intensity variation and lifetime of this sample over 1 s are plotted in Fig. 14(f). Finally, the hippocampal neuron’s fluorescence intensity and lifetime waveforms of single AP and their means [black lines and green line in Fig. 14(g), respectively] were acquired experimentally. This analysis revealed that a single spiking event led to an average relative fluorescence intensity change (ΔF/F) of −2.9% and a lifetime change of −0.7  ns.

### 4.2 Temperature Sensing

Temperature, as an important biomarker, is linked to many biological processes (e.g., metabolism^178^) and medical procedures (e.g., photothermal therapy^179^). Accurate and real-time temperature sensing is important to pathology diagnostics, physiology monitoring, and therapeutical efficiency. Photoluminescence thermometry presents an emerging method by utilizing the temperature-sensitive optical emissions of photoluminescent materials as well as optical detections at high spatial resolution. Its merits include noncontactness, high adaptability to a broad temperature range, high accuracy, flexibility in sample selection, and suitability for diverse environments.^180^ Thus, photoluminescence thermometry is increasingly featured in recent advances in optical temperature measurements.

The success of photoluminescence thermometry depends on two essential constituents: temperature indicators and optical imaging instruments. Recent advances in biochemistry, materials science, and molecular biology have unveiled numerous labeling indicators for photoluminescence thermometry.^162^^,^^181^^,^^182^ From semiconductor quantum dots^183^ and organic fluorophores^184^ to rare-earth-doped phosphors,^185^ the diversity of these agents allows for tailored temperature sensing across different thermal sensitivities, optical properties, and response times for biomedical applications.^186^^–^^189^ For example, lanthanide-doped upconverting nanoparticles (UCNPs), which can sequentially absorb two (or more) low-energy near-infrared photons and convert them to one higher-energy photon, enable biocompatible temperature sensing with low excitation power densities and high sensitivity.^190^^,^^191^

**Fig. 15:**
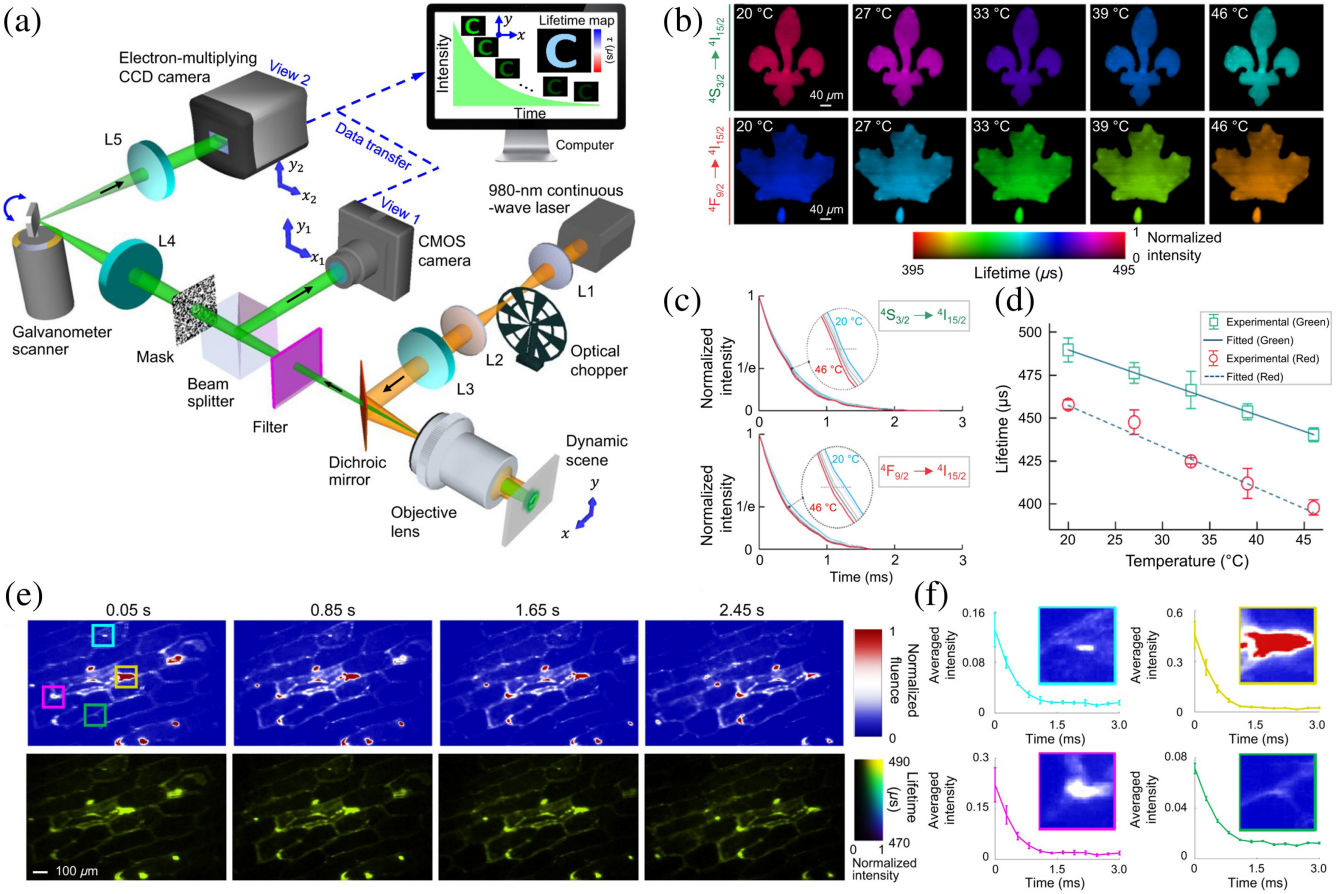
CUP of temperature sensing. (a) Schematic of wide-field photoluminescence lifetime thermometry based on a dual-view rotating-mirror CUP system. L1 to L5, lenses. (b) Lifetime maps of the two emission bands (i.e., S43/2→I415/2 and F49/2→I415/2) of the used UCNPs under different temperatures. (c) Normalized photoluminescence decays of the two emission bands after averaging over the FOV. (d) Temperature-lifetime relationship of both emission bands. (e) Selected time-unsheared views (top row) and reconstructed lifetime maps (bottom row) of a moving onion epidermis cell sample labeled by UCNPs. (f) Intensity decays at four selected areas with different intensities marked in (e). Adapted with permission from Ref. 135.

CUP has enabled wide-field temperature mapping using photoluminescence lifetimes of UCNPs.^135^ In the schematic shown in Fig. 15(a), near-infrared pulses, generated by a 980-nm continuous-wave (CW) laser and an optical chopper, are focused on the back focal plane of an objective lens to form wide-field illumination. The excited UCNPs on the sample emit visible upconversion luminescence. After passing the filter, the dynamic photoluminescence of a selected emission band is imaged by a rotating mirror-based dual-view CUP system at 33,000 fps. The reconstructed lifetime images in the UCNPs’ two upconversion emission bands at different temperatures are shown in Fig. 15(b). The averaged intensity decays [Fig. 15(c)] enable the establishment of the temperature-lifetime relationship [Fig. 15(d)]. Furthermore, the system tracked the 2D temperature of a moving onion epidermis sample labeled by UCNPs at a rate of 20 lifetime-maps per second [Fig. 15(e)]. The intensity decays of four selected areas [labeled in the top-left panel in Fig. 15(e)] are shown in Fig. 15(f). It is worth noting that the fluences of the four selected areas are different but the measured photoluminescence lifetimes remain stable, showing the lifetime-based approach contributed by CUP is more reliable in accurate temperature sensing.

### 4.3 Microfluidics

**Fig. 16:**
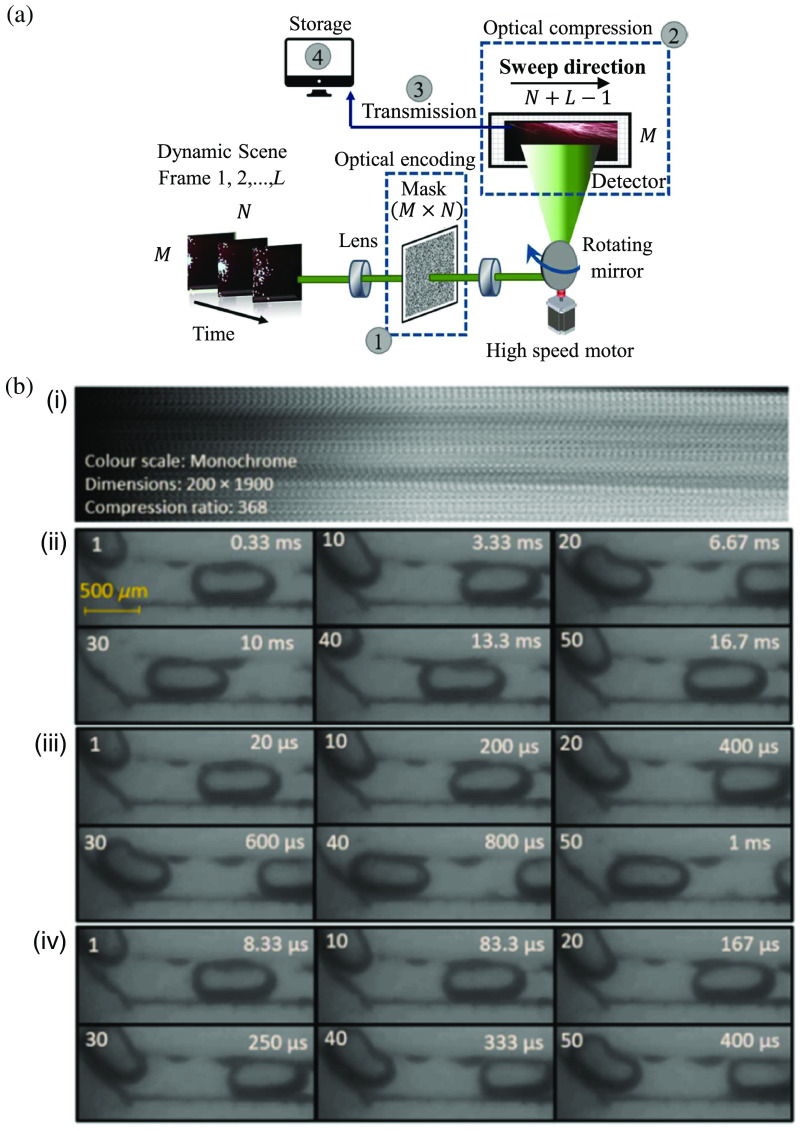
CUP of microfluidics. (a) Schematic of a rotating-mirror-based CUP system. (b) Snapshot of flowing immiscible liquids (i) and representative frames from the reconstructed videos at (ii) 3000, (iii) 50,000, and (iv) 120,000 fps. Adapted with permission from Ref. 148 and Ref. 149.

A rotating-mirror CUP system has been applied to the video recording of complex fluid dynamics and interactions at the microscale.^148^ A schematic of rotating-mirror-based CUP is shown in Fig. 16(a). This system observed flow droplet samples within a microfluidic chip.^149^ Two immiscible liquids of transparent oil and chemical dye, injected through a motorized dispenser, flowed in the chip channels at 0.9  m/s. Three separate measurements are recorded at 3000 fps, 50,000 fps, and 120,000 fps [Fig. 16(b)]. These experimental results show a high reconstruction quality with well-preserved edge features in the frames, showing clear and distinguishable droplets flowing in the microfluidic chip. These results show CUP’s potential to visualize cell-shape changes in response to rapid external stimuli or internal dynamics in microfluidics,^192^^–^^194^ which will provide new insights into cellular biomechanical properties that are closely linked to cellular function and disease development.

### 4.4 Photoacoustic Imaging

**Fig. 17:**
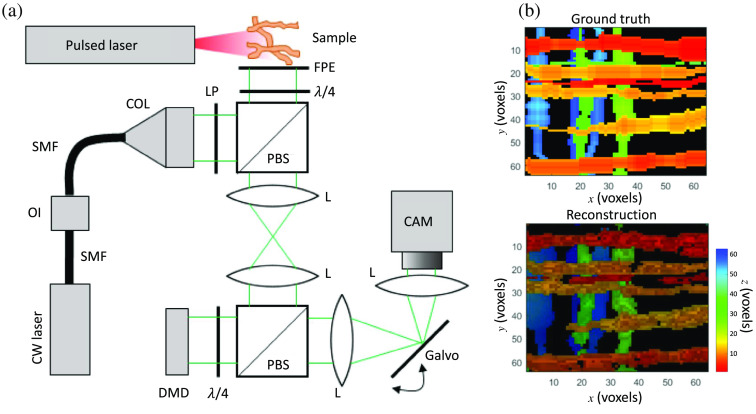
CUP of photoacoustic imaging. (a) Proposed system schematic. CAM, camera; COL, collimator; CW, continuous-wave; DMD, digital micromirror device; FPE, Fabry–Pérot etalon; L, lens; λ/4, quarter wave plate; LP, linear polarizer; OI, optical isolator; PBS, polarizing beam splitter; SMF, single-mode fiber. (b) Simulation of image reconstruction of initial pressure distribution. Adapted with permission from Ref. 195.

CUP can also contribute to photoacoustic (PA) imaging. Figure 17 shows a simulation study on implementing CUP with optical interferometric detection of PA waves.^195^^,^^196^ In the proposed system schematically shown in Fig. 17(a), a pulsed laser illuminates a biological sample. The induced PA effect generates thermoelastic initial pressure, which is detected at the surface of the sample with a Fabry–Pérot etalon (FPE). This interaction of the ultrasonic waves with the surface of the FPE results in the modulation of the reflected CW laser beam on the opposite side of the FPE.^197^ The modulated CW laser beam is then imaged by a CUP system based on a DMD and a galvanometer scanner. Figure 17(b) shows a simulation of this method to image the initial pressure distribution of 12 vessel-like structures.

## 5 Prospect

CUP has largely advanced ultrafast imaging instrumentation. Its generic sensing model indicates that deployed components, rather than the theory, limit the system’s performance. Therefore, CUP has vast potential to be further improved in its imaging capability. In this section, we outline seven aspects of CUP’s future technical development.

### 5.1 Faster

As currently the world’s fastest optical imaging technology, CUP naturally carries a mission to explore even higher speeds in optical imaging. Since CUP’s invention, innovation in temporal shearing units has been a focus of its technical improvement. From $100\times 10^{9}\;\mathrm{fps}$ of the original CUP system,^17^ various image-converter streak tubes have been deployed to increase its imaging speed to $10\times 10^{12}\;\mathrm{fps}$,^114^ which currently holds the world record for single-shot receive-only ultrafast imaging. However, at this speed, the image quality is considerably affected by the space-charge effect,^160^ posing challenges in further improvement of frame rates. In the future, transient perturbation in reflective index induced by a temporally modulated ultrashort laser pulse or molecular orientation could bring higher imaging speeds and circumvent image degradation.^151^^,^^170^

Leveraging the advance in chirped pulse illumination, CUP systems using passive temporal shearing units have boosted the imaging speed to $3.85\times 10^{12}\;\mathrm{fps}$,^121^ $70\times 10^{12}\;\mathrm{fps}$,^57^ $219\times 10^{12}\;\mathrm{fps}$,^198^ and $256\times 10^{12}\;\mathrm{fps}$.^63^ The last value marks the fastest speed in single-shot optical imaging. In the future, by synergizing the ultra-broadband ultrashort pulses^199^ and photonic streaking in gas,^170^^,^^200^ CUP’s imaging speed could top quadrillion fps, entering the attosecond-level imaging regime.

### 5.2 Clearer

A higher spatial resolution allows CUP to visualize fine details. In biomedicine, this ability can transfer to informative depiction of cellular and tissue morphology, accurate diagnostics, and precise treatment. Nonetheless, in CUP’s operation, both spatial encoding in data acquisition and denoising in image reconstruction could reduce the effective system bandwidth. To visualize the targeted spatial details, a common practice is to magnify the scene, which unavoidably reduces the FOV. Thus, how to regain the lost bandwidth to achieve a diffraction-limited spatial resolution is an important research direction of CUP. One potential approach is subpixel shifting.^201^ In particular, a DOE could be used to duplicate the dynamic scene to multiple bands, each would be encoded with the same encoding mask but with a different subpixel shift. A joint image reconstruction using all the captured snapshots could recover the original optical bandwidth.

Another interesting research direction is super-resolution CUP. As many ultrafast phenomena also occur at the nanoscale, overcoming the diffraction limit in the CUP system will likely open avenues for new studies not possible before, including temperature dynamics in mitochondria,^202^ conformational transitions of protein,^203^ evolutions of membrane fragments produced by cellular lysis.^204^ Toward this goal, CUP can be incorporated into existing super-resolution microscopy techniques (e.g., structured illumination microscopy^205^) or bypass the optical diffraction limit by electron imaging (e.g., transmission electron microscopy^117^).

### 5.3 Broader Spectrum

Although CUP has been experimentally demonstrated in the ultraviolet, visible, and near-infrared spectral ranges, extending its imaging capability to a broader spectrum will likely continue in future application-driven development. Toward this goal, the spatial encoder should have high contrast in the desired spectrum. Besides the popular broadband metallic masks made from aluminum, silver, or chromium,^17^^,^^91^^,^^147^ photonic crystals with broad tunable bandgaps can selectively block specific wavelengths,^206^ giving them the potential to be used as a spatial encoder in certain spectra. For temporal shearing units, the photocathode in the image-converter streak tube excludes high-sensitivity imaging for wavelengths of >950  nm. Contrarily, leveraging its all-optical functionality, rotating mirrors can fill out this gap, which will likely lead to the development of CUP for deep ultraviolet, mid-infrared, and far-infrared spectra. Moreover, advanced design and fabrication of metasurfaces and metalenses could potentially extend CUP to a spectrum from extreme ultraviolet to terahertz.^207^^,^^208^

### 5.4 Smarter

Many deep learning-based approaches have been used in CUP’s image reconstruction.^94^^–^^104^ Harnessing the power of artificial intelligence, they have unlocked new capabilities for analyzing ultrafast events, such as real-time data processing, on-device analysis, and on-time feedback. It is expected that these deep-learning algorithms will provide multifacet supervision to CUP systems in the future. For example, the next-generation systems could autonomously adjust the patterns loaded on the spatial encoder according to the initial classification of the dynamic scene. These systems could also monitor the nonlinear shearing operation and adaptively compensate for it in image reconstruction or system alignment.^104^

### 5.5 Higher Dimensions

Recent developments in CUP have explored high-dimensional ultrafast imaging. To date, several advanced systems—such as multispectral CUP,^120^ stereo-polarimetric CUP,^57^ and spectral-volumetric CUP^62^—have pushed the overall sensing capability to four dimensions and even five dimensions. In the future, by extending the configuration used in stereo-polarimetric CUP^57^ to generate multiple perspectives of the dynamic scene, light-field imaging could be incorporated into CUP. Ultimately, single-shot imaging of seven-dimensional plenoptic function would be within reach. Using CUP to sense other photon tags that are not included in the conventionally defined plenoptic function is also a future direction. CUP has already enabled amplitude and phase imaging of a femtosecond laser pulse.^63^ CUP could also be combined with other existing technologies, such as the transport-of-intensity equation ^209^ and coherent modulation,^210^ for ultrafast quantitative phase imaging. Finally, recent advancements in on-chip polarization imaging and metasurface-based angular moment separation could incorporate these parameters into CUP’s measurement scope.^211^^,^^212^

### 5.6 Smaller

CUP systems with a compact size are important to studies that require restricted weight and space. For biomedicine, it will offer the ability to mount the system the same way as conventional cameras on microscopes and hand-held systems as well as in operating rooms. An innovative optical design that folds the optical path could reduce the system size.^213^ Selecting a multifunctional component (e.g., a metalens and a TDI sensor) provides another approach to reducing the number of optical elements in CUP systems. Advances in sensor design and nanofabrication could provide the streak imaging sensor^167^^,^^168^ with a 2D FOV. All efforts will contribute to engineering compact and even miniature CUP systems in the future.

### 5.7 Cheaper

Besides reducing the size of a CUP system, making an economical CUP system carries considerable value from both research and commercialization perspectives. To manufacture a fixed spatial encoder, high-resolution printing offers the lowest cost (i.e., <$20 per ${\mathrm{in.}}^{2}$). For reconfigurable spatial encoders, a 0.47″ DMD chip (1920×1080 micromirrors; 5.4-μm pitch) costs ∼$120.^214^ Future development could develop a DMD controller specifically tailored for CUP with much-reduced functionality compared with existing ones to decrease the cost. For the temporal shearing unit, the first approach to reduce the cost is to find a replacement for expensive image-converter streak tubes. Electro-optic modulators have made their debut in this direction,^122^ which produced an imaging speed of $50\times 10^{9}\;\mathrm{fps}$. For a rotating-mirror CUP system, a viable strategy is to add the spatial encoder and an affordable rotating mirror (e.g., galvanometer scanners and polygonal mirrors) in front of the CCD/CMOS cameras existing in the system. It is envisaged that a minor addition in cost could endow ultrahigh-speed imaging to existing cameras while retaining their inherent advantages (e.g., in sensitivity and sensing spectrum).

## 6 Conclusions

In this tutorial, we have elucidated the fundamentals of CUP. We have provided Matlab codes that create CUP’s sensing matrices and simulate the acquired snapshots based on the forward model. Matlab/Python codes and examples are also included for two respective reconstruction algorithms—one based on analytical modeling using the ADMM and the other on deep learning using the D-HAN. To facilitate comprehension, a “cell-division” scene is simulated step by step alongside the provided codes. A fully operational CUP system relies on three essential hardware components: a spatial encoder, a temporal shearing unit, and a 2D sensor. We have surveyed representative implementations of each component as well as calibration steps in both data acquisition and image reconstruction.

Ever since its invention, CUP has stayed under the spotlight in research as an emerging and innovative imaging platform. Its evolution has been shaped by the innovation of imaging strategies and the adaptive optimization of its key components, leading to its widespread implementation in various biomedical applications. CUP—as currently the world’s fastest single-shot optical imaging modality—is positioned for future advancements in imaging speed, spatial resolution, sensing spectrum, artificial intelligence, imaging dimensionality, system size, and manufacturing cost. CUP is highly anticipated to make more remarkable progress in biomedicine.

## Data Availability

All data and software in support of this work are available in the manuscript and can be downloaded from Refs. 68, 92, 111, and 112.
